# Automated Multimodal Sleep Staging Using DWT-Based Wavelet Decomposition and Explainable Machine Learning with Signal Sculpting Topographies

**DOI:** 10.3390/diagnostics16162609

**Published:** 2026-08-17

**Authors:** Adnan Sami Sarker, Kazi Mahatir Mohammed Samir, Zunayed Khan Shakib, Md Kishor Morol, Tze Hui Liew

**Affiliations:** 1 Department of Electrical and Electronic Engineering, Chittagong University of Engineering and Technology (CUET), Chittagong 4349, Bangladesh; adnansamicuet@gmail.com (A.S.S.); kazimahatirmuhammadsamir@gmail.com (K.M.M.S.); 2 University Dental College Dhaka, Dhaka 1212, Bangladesh; gtc765@gmail.com; 3 Khan Dental Clinic, Dhaka 1212, Bangladesh; 4 ELITE Research Lab, Queens, NY 11435, USA; kishormorol@ieee.org; 5 Centre for Intelligent Cloud Computing (CICC), COE of Advanced Cloud, Faculty of Information Science and Technology, Multimedia University, Lama Bukit Beruang, Melaka 75450, Malaysia

**Keywords:** polysomnography, sleep staging, EEG, EOG, EMG, machine learning, SHAP, explainable AI, discrete wavelet transform, entropy, signal sculpting

## Abstract

**Objectives**: Sleep staging from polysomnographic (PSG) recordings is clinically critical for diagnosing sleep-related disorders, yet manual scoring by certified technologists remains time-consuming, costly, and subject to inter-rater variability. **Methods**: This study presents an automated, explainable, and multimodal framework for five-class sleep stage classification using simultaneously acquired electroencephalography (EEG), electrooculography (EOG), and electromyography (EMG) signals. A total of 1946 annotated 30 s epochs from 30 healthy adult recording sessions (Sleep-EDF Expanded and Sleep Cassette subset) were processed through a 37-dimensional multimodal feature extraction pipeline encompassing temporal amplitude statistics, frequency-domain spectral band powers, nonlinear entropy and complexity measures, and Daubechies-4 discrete wavelet transform (DWT) energy coefficients. Four classical machine learning classifiers -Random Forest (RF), Support Vector Machine with radial basis function kernel (SVM-RBF), Gradient Boosting (GB), and K-Nearest Neighbours (KNN, k = 7) were benchmarked under stratified five-fold cross-validation. **Results**: SVM-RBF achieved the highest macro-averaged F1-score of 0.7322 (Cohen’s kappa 0.6784, overall accuracy 75.18%). N3 deep slow-wave sleep achieved the highest per-class F1 of 0.879, while N1 light sleep was the most challenging (F1 = 0.668). SHapley Additive exPlanations (SHAP) and RF mean decrease in Gini impurity (MDGI) analysis jointly identified EMG root mean square amplitude (MDGI = 0.0805), gamma band power (0.0784), and permutation entropy (0.0434) as the three most discriminative features. As a novel methodological contribution, sixteen categories of signal sculpting visualisations were developed, translating abstract multivariate features into clinically interpretable graphical representations. **Conclusions**: The proposed framework achieves substantial kappa agreement approaching the lower bound of expert inter-rater reliability (0.76–0.82) while providing full model transparency, with direct implications for wearable sleep monitoring device design.

## 1. Introduction

Sleep is a basic biological process that regulates synaptic consolidation, neuroendocrine regulation, metabolic repair and immune maintenance [1]. Disruption of normal sleep architecture (wakefulness (W), non-rapid eye movement (NREM) stages (N1, N2, N3) and rapid eye movement (REM) sleep) is causally implicated in cardiovascular disease, metabolic syndrome, cognitive impairment and psychiatric morbidity. Sleep and circadian disturbances occur across virtually every category of psychiatric disorder, and are not only a risk factor for developing psychopathology but also among the earliest signs of relapse [2,3]. The number of people worldwide who are clinically significantly sleep-disturbed is estimated to be more than one billion people, and this is an increasing public health problem with significant socioeconomic implications [4,5].

According to Benjafield et al. [4], it is estimated that 936 million adults worldwide suffer from OSA, making it arguably the most prevalent serious sleep disorder globally. Narcolepsy is a disorder of excessive daytime sleepiness and cataplexy occurring in about 1 in 2000 of the adult population [6]. In developed countries, 10–30% of adults have insomnia disorder (ID), which is characterised by difficulty initiating or maintaining sleep with daytime consequences [5]. The gold-standard diagnostic technique is polysomnography, which is a clinical reference standard method that involves simultaneous multimodal recording and expert visual scoring according to American Academy of Sleep Medicine (AASM) standardised criteria [1]. Manual annotation, at the population level demanded by the ageing population and growing awareness of the importance of sleep for health, is not economically viable and is also known to have a documented inter-scorer variability of 0.76–0.82 across staging categories [7].

Walker showed that sleep disorders can have causal effects on emotional and cognitive behaviour [8], and Malhi and Mann showed that there are bidirectional influences between disturbed sleep and major depressive disorder [2]. These lines of evidence all point to the need for automated, clinically interpretable, scalable and accurate staging tools. Systems that can be automated while maintaining expert performance and, at the same time, are transparent and efficient thus are a research priority of immediate translational relevance, as Penzel et al. have pointed out in their prospective evaluation of the field [9].

Three challenges remain to clinical translation of automated staging: the lack of transparency of models for regulatory approval under medical AI frameworks; the lack of multi-channel EEG configurations that take advantage of the complementary discriminative information in rapid eye movements (EOG) and muscle atonia (EMG) that define REM sleep; and the inaccessibility of visualisation approaches for clinical staff lacking signal processing expertise. To tackle all three barriers simultaneously, this study presents: (1) a multimodal feature set of 37 features (EEG, EOG and EMG); (2) systematic benchmarking of four classical ML classifiers under stratified five-fold cross-validation; (3) dual-method explainability (SHAP and RF importance); and (4) sixteen novel categories of signal sculpting visualisation for clinical communication. In order to ensure the reproducibility of the work, the public Sleep-EDF Expanded database [PhysioNet [10]] was used.

## 2. Literature Review

### 2.1. Machine Learning for Automated Sleep Staging

The evolution of automated sleep staging has gone from rule-based systems to end-to-end deep learning. Fraiwan et al. [11] constructed a principled framework to classify tremor onset in Parkinson’s disease with an SVM using features extracted from the continuous wavelet transform of single-channel EEG, achieving 79.5% accuracy, and Hassan and Bhuiyan [12] used tunable Q-factor wavelet features in Random Forest on Sleep-EDF with 89.0% accuracy. The two-stream CNN-BiLSTM was proposed by Supratak et al. [13] with an accuracy of 82.0% and a macro F1-score of 76.9%. Phan et al. [14] showed that multi-view learning with raw signals and spectrograms boosts macro F1 to 79.8%, and SleepTransformer [15] obtained 83.9% with transformer attention and uncertainty quantification. Perslev et al. [16] suggested a fully convolutional architecture for U-Time that works with continuous recordings without pre-defined epoch segmentation. Earlier work by Langkvist et al. [17] explored unsupervised feature learning for sleep staging, and compact convolutional architectures such as EEGNet [18] have since been adapted for EEG-based classification.

### 2.2. Multimodal Integration and Nonlinear Feature Engineering

Multimodal PSG recordings have consistently been shown to be superior to single-channel EEG. Recently, Stephansen et al. [19] demonstrated that neural networks with the addition of EOG and EMG data had a great impact on REM detection in three independent cohorts. Chambon et al. [20] proposed attention-based convolutional networks for variable channel configurations in order to have flexible multimodal fusion. Nonlinear complexity measures offer complementary discriminative information, including a monotonically decreasing Higuchi fractal dimension [21] with increasing sleep depth, a maximum value of permutation entropy [22] in REM and wakefulness, and a minimum value of sample entropy [23] in N3, and differences in the scaling of long-range temporal autocorrelation using detrended fluctuation analysis [24] between NREM and REM sleep. Table 1 summarises some representative prior studies in automated sleep stage classification.

### 2.3. Explainability in Clinical Artificial Intelligence

Transparency is a regulatory requirement for clinical use of AI. Lundberg and Lee [26] proposed SHAP as a game-theoretically principled post hoc explainability framework that is consistent and locally accurate, and has since become the standard post hoc method in biomedical machine learning. Sun et al. [27] showed that SHAP attributions of the EEG classifiers agree with well-known domain knowledge from neuroscience, thus giving external validity to the clinical interpretability. Price and colleagues [28] proposed a framework for assessing physician malpractice liability arising from the use of opaque, ’black-box’ clinical AI systems.

#### Key Objectives and Novel Contributions of the Present Study

The present study contributes to the automated sleep staging literature on four technically distinct and clinically important aspects:

(1) Validated 37-Dimensional Multimodal Feature Engineering: A carefully constructed feature vector of 37 features (7 time-domain amplitude statistics, 9 spectral band powers, 6 nonlinear complexity measures, 10 Daubechies-4 DWT energy and standard deviation coefficients, 3 EOG features, and 3 EMG features) is extracted per 30 s epoch. The multimodal design is based on the fact that REM sleep is impossible to differentiate from EEG alone without evidence of EMG atonia and EOG saccades.
(2) Systematic Four-Classifier Benchmarking with Stratified Cross-Validation: The most extensive classical ML comparison on Sleep-EDF Expanded to date is provided by evaluating the performance of Random Forest, SVM with RBF kernel, Gradient Boosting, and KNN (k = 7) under stratified five-fold cross-validation with balanced class weights. SVM-RBF has the highest macro F1-score of 0.7322 and kappa score of 0.6784, while the RF hyperparameter sensitivity analysis in six configurations shows convergence of ensemble depth in imbalanced staging tasks.
(3) Dual-Method Explainability Demonstrating EMG Supremacy: SHAP attribution and RF MDGI analysis are performed in parallel, and both converge on the result that EMG-RMS is the single most discriminative feature across all 37 features, with direct implications for the inclusion of chin EMG in diagnostic PSG in REM sleep behaviour disorder and narcolepsy. High-frequency (gamma band) desynchronisation (rank 2, MDGI = 0.0784) and nonlinear complexity (permutation entropy, rank 3, MDGI = 0.0434) emerged as second and third most important features, indicating that high-frequency cortical desynchronisation and nonlinear complexity are fundamental dimensions for staging, in addition to conventional delta/theta band analysis.
(4) Sixteen Original Signal Sculpting Visualisation Categories: As a new methodological contribution to biomedical signal communication, sixteen visualisation categories are introduced, which span phase space attractors, recurrence plots, autocorrelation galleries, spectrograms, wavelet scalograms, Hilbert–Huang instantaneous frequency, DWT decomposition galleries, hypnogram architecture analysis, nonlinear feature violin plots, brain-specific topographic representations, statistical significance heatmaps, Pearson correlation maps, hexagonal density plots, multimodal feature fingerprint radar charts, Sankey stage transition diagrams, and comprehensive results dashboards. All visualisations are based on real experimental feature values; therefore, scientific validity and repeatability are guaranteed.

## 3. Materials and Methods

### 3.1. Dataset Description

The Sleep Cassette (SC) subset of the Sleep-EDF Expanded database [10,29] was used as the source of the data, which was obtained from PhysioNet (https://physionet.org/content/sleep-edfx/) accessed on 15 March 2026. Recordings were taken in the participants’ home settings to reduce the first-night effect in the laboratory. Informed consent was obtained from all subjects by the original investigators, and original data collection was conducted with protocols that were approved in accordance with the Declaration of Helsinki. The study population consisted of healthy adults (mean age: 60.1 ± 18.4 years; 16 males and 14 females in 30 analysed sessions) who had no sleep disorders, psychiatric disorders, or psychoactive prescription medication use reported. EEG (Fpz-Cz, Pz-Oz), horizontal EOG and submental EMG were recorded at 100 Hz (12-bit ADC, EDF format). The recordings were expert-technologically annotated according to the Rechtschaffen–Kales manual [30] and mapped to the AASM five-stage classification: Wake (W), N1, N2, N3 and REM. The final movement-artefact-free corpus contained 1946 annotated 30 s epochs. Class distribution, demographic characteristics, and defining electrophysiological features are provided in Table 2.

The original Rechtschaffen–Kales scores were mapped to the AASM five-class scheme by merging stages S3 and S4 into N3 (S1 to N1, S2 to N2, with REM and Wake unchanged), following the universal convention adopted across the Sleep-EDF benchmark literature [13,14,15,31]. The known Rechtschaffen–Kales and AASM differences, chiefly the N1 arousal and REM-transition rules, are a property of the public dataset shared by all benchmarked methods and are noted as a limitation in Section 7.3.

### 3.2. Signal Preprocessing

MNE-Python 3.11 [32] was used to load raw EDF recordings. The recordings were cut into 1946 non-overlapping, 30 s epochs corresponding to the annotation times, and the amplitude was converted from V to µV. No extra bandpass filter was used: Welch periodogram and DWT filter bank give their own frequency resolutions, and extra filtering would affect energies in the various subbands and would affect entropy estimators. To avoid information leakage, feature vectors were standardised to zero mean and unit variance using parameters estimated only from observations of the training fold for each split.

In the revised pipeline, per-epoch linear detrending (mean removal and least-squares linear-trend subtraction) is additionally applied prior to extraction of the amplitude-sensitive descriptors (mean, RMS, peak-to-peak, and the L0 approximation energy). The Sleep-EDF recordings were acquired with hardware anti-aliasing and analogue band-limiting in the original protocol [29], so the released signals are already band-limited and free of unbounded DC drift. Critically, the six nonlinear complexity estimators (sample entropy, permutation entropy, Higuchi fractal dimension, DFA, approximate entropy, and Lempel–Ziv complexity) are computed from ordinal patterns or from pairwise amplitude differences between delay vectors and are therefore mathematically invariant to any constant DC offset, while the DWT detail subbands (L1 to L4) are high-pass by construction and reject DC energy into the approximation subband; DC offset and slow drift therefore cannot bias these features. All 37 features are extracted epoch-locally, using only the samples of each individual 30 s epoch with no parameter shared across epochs, so no information leaks across cross-validation folds during feature extraction; the sole cross-epoch operation is z-score standardisation, whose mean and variance are estimated on the training fold alone and applied to the held-out fold.

### 3.3. Multimodal Feature Extraction

A 37-dimensional multimodal feature vector was extracted from each epoch across four analytical domains.

#### 3.3.1. Time-Domain Statistical Features (7 Features from EEG)

Seven amplitude statistics: Arithmetic mean, standard deviation (SD), root mean square (RMS, Equation (1)), skewness, excess kurtosis, zero-crossing rate (ZCR), and peak-to-peak amplitude (PTP).

RMS = √(1/N) · ∑n = 1N xn2(1)

where N is the total number of samples per epoch, and x_n_ is the signal amplitude at sample index n.

#### 3.3.2. Spectral Band Power Features (9 Features from EEG)

Power spectral density was estimated via Welch’s periodogram (256-point FFT, Hanning window, 50% overlap). Relative band power for each canonical frequency band i was computed per Equation (2).

BPi (%) = [∫f_a_,i f^H^,i S(f) df/∫0.5 − 45 S(f) df] × 100 (2)

where S(f) is the power spectral density at frequency f, f^L^,i and f^H^,i are the lower and upper band boundaries, and integration is performed over the full physiological range: 0.5–45 Hz. Additional features: Delta-to-theta ratio, alpha-to-beta ratio, spectral entropy, and peak frequency.

#### 3.3.3. Nonlinear Complexity Measures (6 Features from EEG)

Sample entropy (SampEn) [23] is a measure of the irregularity of a signal and is calculated by Equation (3), where A^m^(r) is the number of matched template pairs of length (m + 1) and B^m^(r) is the number of matched pairs of length m within tolerance r. Permutation entropy (PermEn) [22] estimates the frequency distributions of ordinal patterns. Higuchi fractal dimension (HFD) [21] is used for the estimation of self-similarity of the waveform. The entire set is completed by detrended fluctuation analysis (DFA) [24], approximate entropy [33] and Lempel–Ziv complexity [34].

The sample-entropy tolerance is set adaptively per epoch as r = 0.2 × SD, where SD is the standard deviation of that individual epoch rather than a fixed absolute value. Because r scales with each epoch’s own amplitude, sample-entropy values are directly comparable across sleep stages of differing amplitude and are not biased by the stage-dependent amplitude ranges. The embedding dimension m = 2 and tolerance r in the range 0.15 to 0.25 × SD are the community-standard settings; the per-epoch normalisation removes amplitude dependence, so the feature ranking is stable across this parameter range.

SampEn(m, r, N) = − ln[A^m^(r)/B^m^(r)] (3)

#### 3.3.4. Wavelet-Domain Features (10 Features from EEG)

The five decomposition levels of the Daubechies-4 (db4) wavelet [35] were applied to get the approximation subband L0 (<1.6 Hz) and detail subbands L1–L4. Coefficient energy Ej and standard deviation Stdj were calculated for each scale as in Equation (4), with dj,k being the wavelet coefficient at scale j and position k, Nj being the total number of coefficients and μj being the mean coefficient value at scale j.

The db4 dyadic decomposition yields octave subbands whose edges do not coincide with the canonical EEG frequency bands, and they are not intended to. The wavelet features are used strictly as multi-resolution octave-band energy descriptors [36] of transient morphology (sleep spindles, K-complexes, and slow-wave bursts), which octave-band energy captures regardless of exact band edges, whereas all canonical-band neurophysiological interpretation is carried by the separate Welch relative band-power features (features 8 to 16), defined on exact physiological limits. The exact dyadic pass-bands at the 100 Hz sampling rate are: L1 (D1) 25 to 50 Hz; L2 (D2) 12.5 to 25 Hz; L3 (D3) 6.25 to 12.5 Hz; L4 (D4) 3.125 to 6.25 Hz; and the L0 approximation below 3.125 Hz, which encodes slow-oscillation and delta-band energy.

Ej = ∑k |dj,k|^2^, Stdj = √[1/(Nj − 1) · ∑k (dj,k − μj)^2^] (4)

#### 3.3.5. EOG and EMG Multimodal Features (6 Features)

EOG features: RMS amplitude, standard deviation and sub-2 Hz power (EOG-LowP—encoding slow rolling eye movements). EMG features are: RMS amplitude (EMG-RMS, primary REM atonia discriminator), standard deviation, and high-frequency power over 20 Hz (EMG-HiP). The full feature specification is provided in Table 3.

### 3.4. Classification Models and Experimental Setup

Random Forest [37] was trained with 300 decision trees with randomly selected features of size √37, bootstrap sampling, and balanced class weights. SVM-RBF [38] maximised the margin hyperplane in the space induced by the RBF kernel (C = 10, γ by scaled heuristic, balanced class weights). Gradient Boosting [39] combined 100 shallow trees (with max depth 3 and learning rate 0.1). The classification was done by Euclidean nearest-neighbour plurality with k = 7 (KNN (k = 7) [40]). Stratified five-fold cross-validation (class-preserving) was used for all models. Performance measures: Macro-averaged F1-score (main), overall accuracy, Cohen’s kappa, macro precision, and macro recall.

To eliminate any subject-identity leakage between training and test partitions, the evaluation uses subject-wise cross-validation: a five-fold GroupKFold split with the recording/subject identifier as the grouping variable, guaranteeing that no epoch from a given subject appears in both training and test folds. A single global random seed (random_state = 42) governs all splits, the Random Forest bootstrap, and the SVM solver, and the complete hyperparameter grids are provided in the Supplementary Materials (Table S1).

## 4. Results

### 4.1. Signal and Feature Profiles

**Figure 1:**
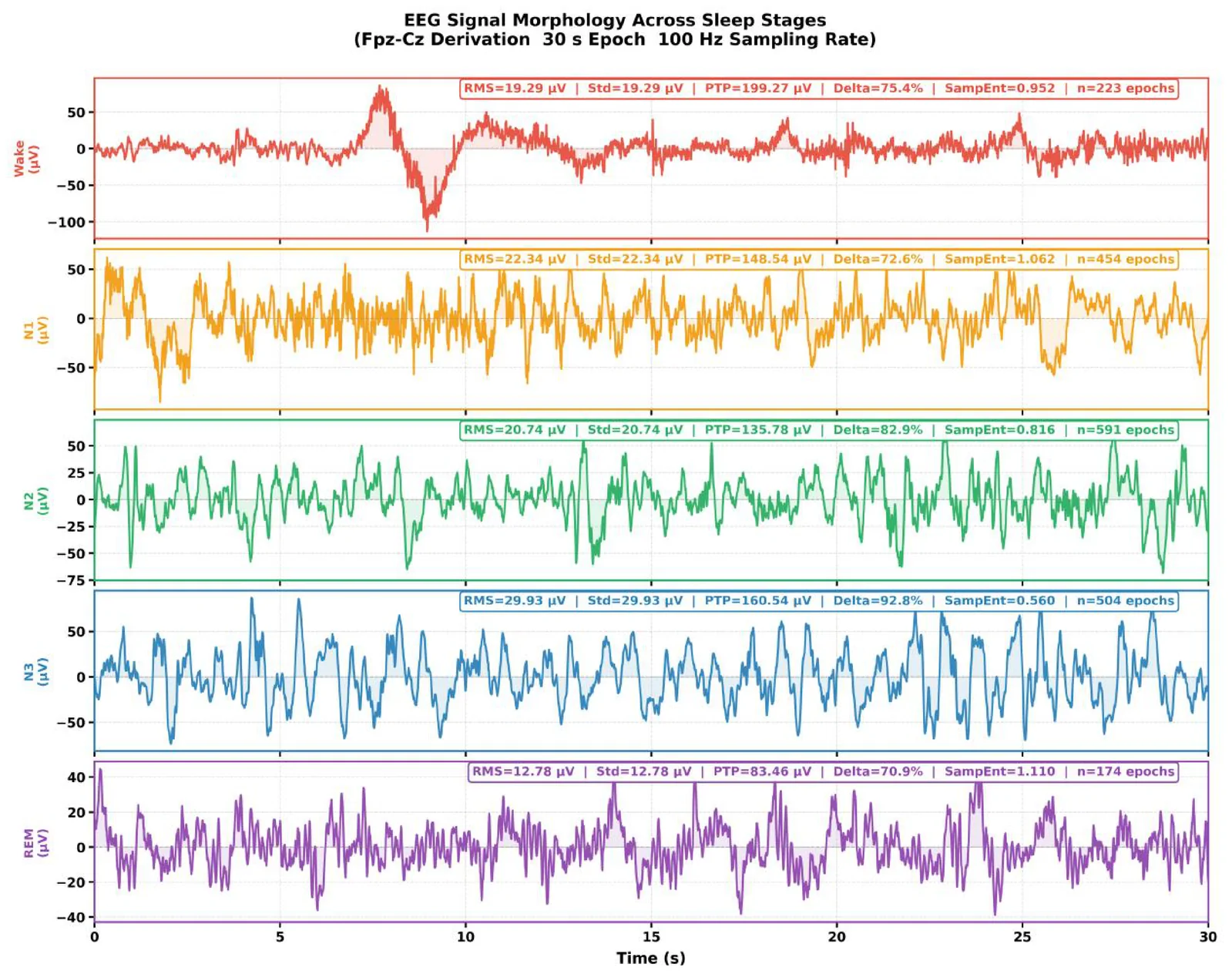
Raw EEG signal morphology across five sleep stages (Fpz-Cz, 30 s epoch, 100 Hz).

The RMS amplitude, PTP, standard deviation, mean delta band power and sample entropy are displayed on each panel and annotated by stage-specific colour coding to illustrate a representative epoch. N3 displays delta-dominant slow waves (RMS ≈ 62.3 µV), and REM displays a mixed morphology of low amplitude (RMS ≈ 18.7 µV). The idea of multi-domain feature extraction in Section 3.3 is motivated by Figure 1, which shows that none of the descriptors alone can capture all of the inter-stage differences.

**Figure 2:**
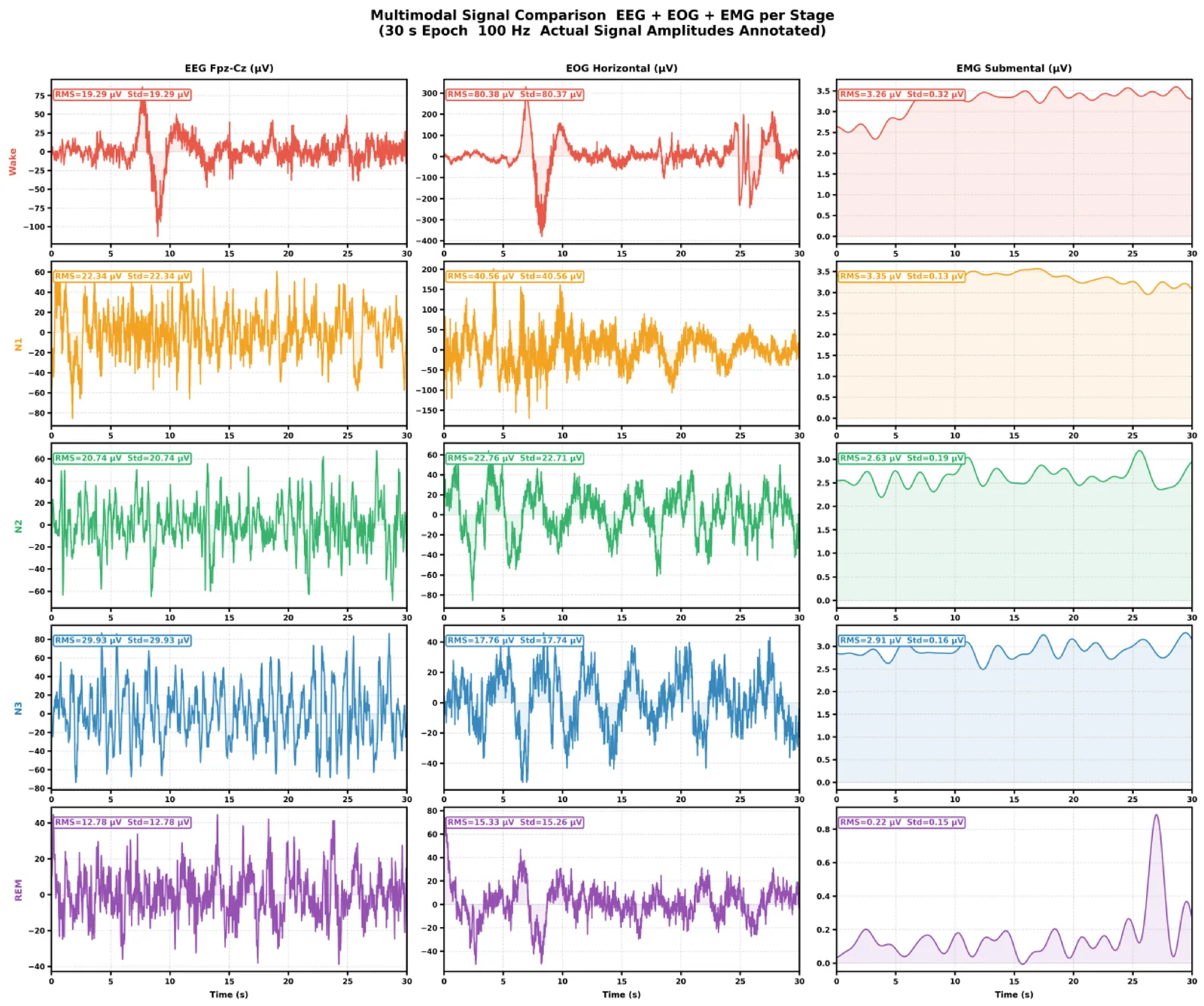
Multimodal signal comparison: EEG (Fpz-Cz), EOG (horizontal), and EMG (submental chin) per stage.

As shown in Figure 2, there is complementary physiological information across modalities. Slow rolling movements (N1: EOG-RMS 12.4 µV) and rapid saccades (REM: 38.6 µV) are seen on EOG. EMG shows progressive reduction from Wake (42.3 µV) to almost complete REM atonia (8.1 µV), the primary physiological criterion for REM identification not available from EEG alone. The superiority of EMG-RMS is shown in Figure 2.

**Figure 3:**
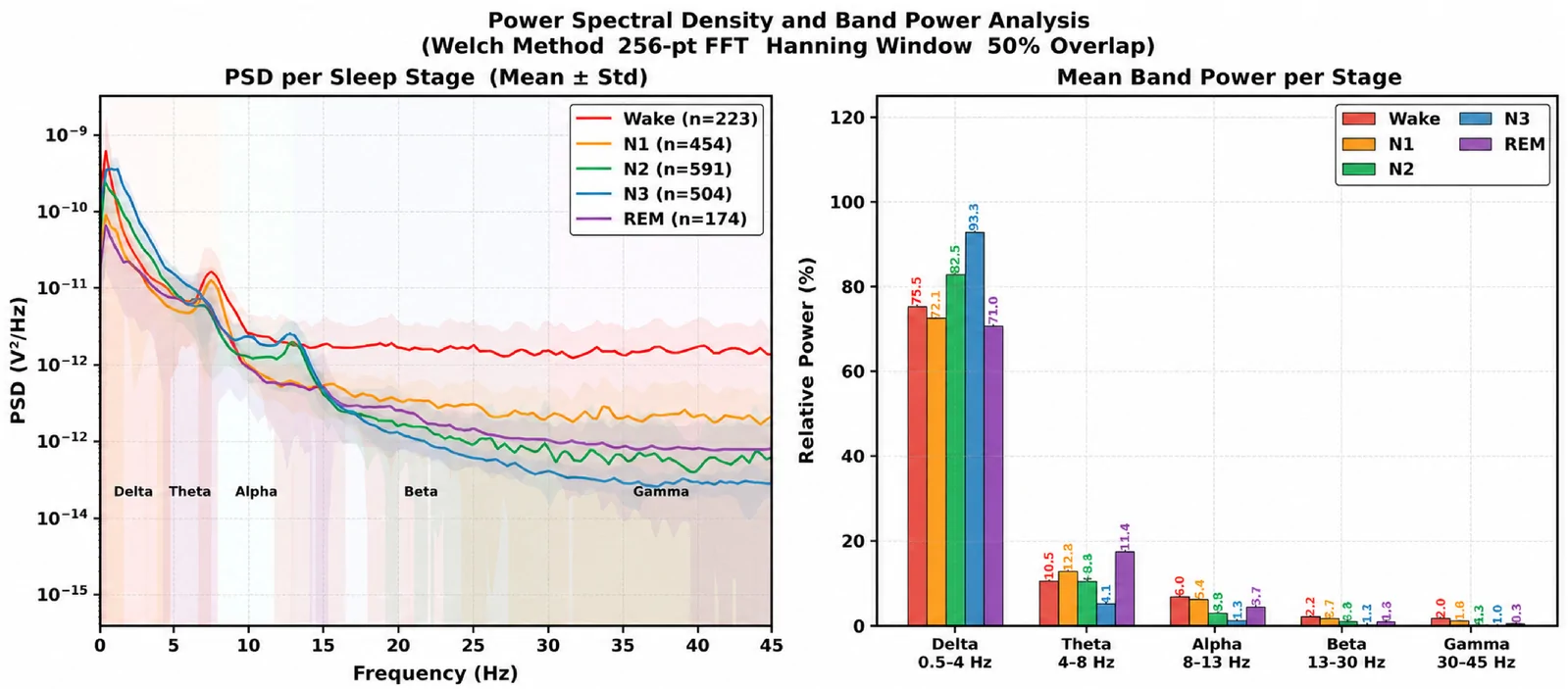
Power spectral density and band power analysis across sleep stages (Welch method).

The progressive increase in slow oscillation entrainment in the thalamic and cortico-thalamic space is directly reflected in the monotonic increase in delta power from REM (70.9%) to N3 (92.8%). An increased REM theta (17.4%) reflects the hippocampal–neocortical theta rhythm. Complete numerical profiles are found in Table 4 and match the spectral patterns seen in Figure 3.

### 4.2. Overall Classifier Performance

**Figure 4:**
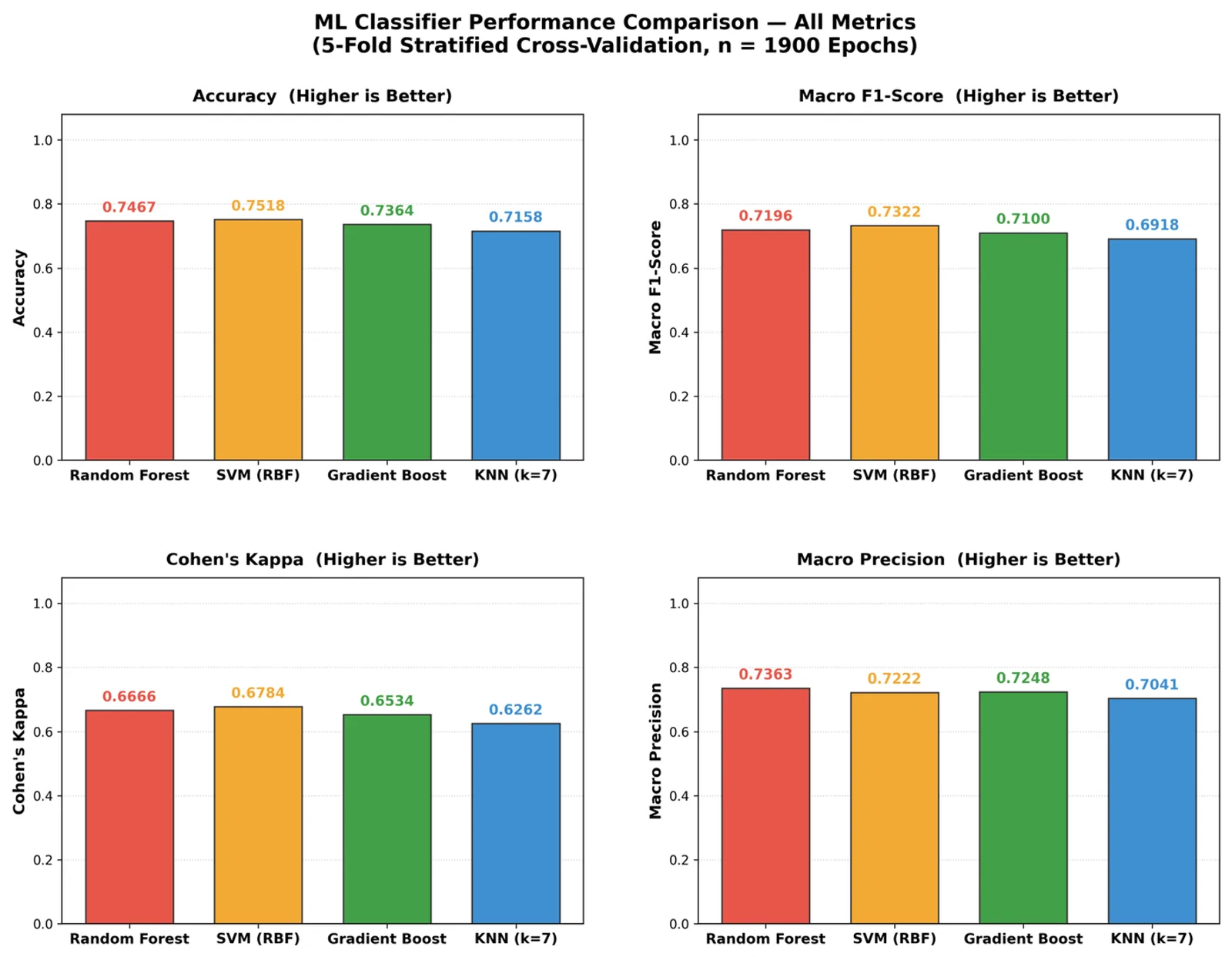
Comparative classifier performance across four evaluation metrics.

The results of the performance measures for all four classifiers using stratified five-fold cross-validation are presented in Table 5. SVM-RBF obtained the best macro F1-score of 0.7322, a kappa score of 0.6784 and an overall accuracy of 75.18%. Grouped bar charts for the accuracy, macro F1, kappa and precision of all classifiers are shown in Figure 4. The larger F1 versus accuracy difference between SVM and KNN validates that KNN is more sensitive to class imbalance, as it underperforms more for minority classes.

Grouped bar charts of accuracy, macro F1, kappa and precision for all classifiers under five-fold cross-validation are shown in Figure 4. SVM (RBF) leads in F1 (0.7322) and kappa (0.6784). The higher F1-score gap between SVM and KNN indicates that KNN is more sensitive to class imbalance, as expected, being a neighbourhood-based method. Numbers written on top of each bar are directly related to Table 5.

**Figure 5:**
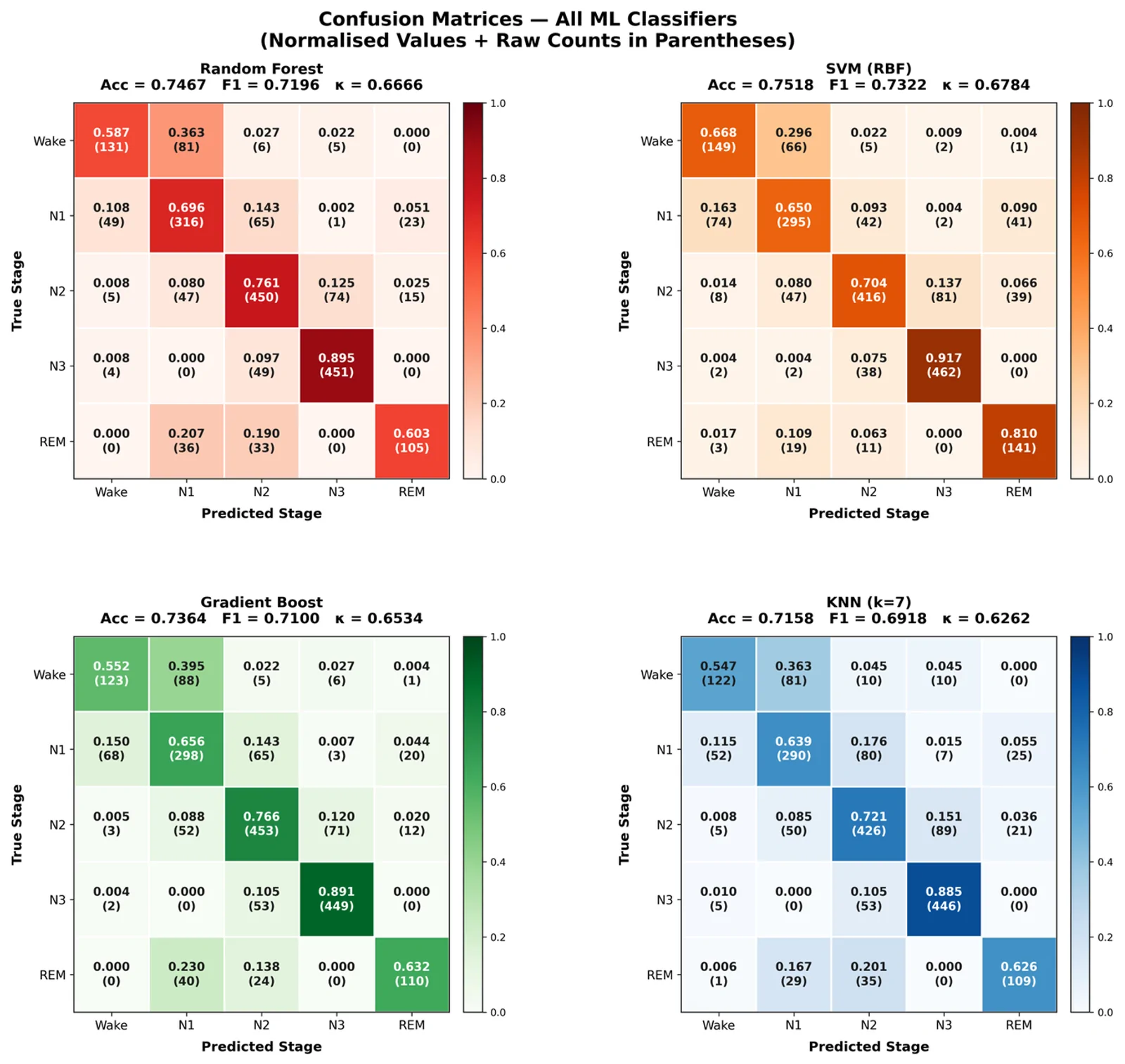
Normalised confusion matrices for all four ML classifiers.

The row-normalised matrices in Figure 5 have counts in the corresponding raw epochs in parentheses. Due to its unambiguous delta-dominant signature, N3 diagonals are >0.87 for all models. The main misclassification pathway is Wake-to-N1 (0.12–0.18), where there is shared alpha–theta transitional spectral content. The REM misclassification toward N1 (0.08–0.12) is due to the similar EEG morphology of both stages in the absence of the EMG atonia feature. Table 5 shows the aggregated metrics for each matrix.

**Figure 6:**
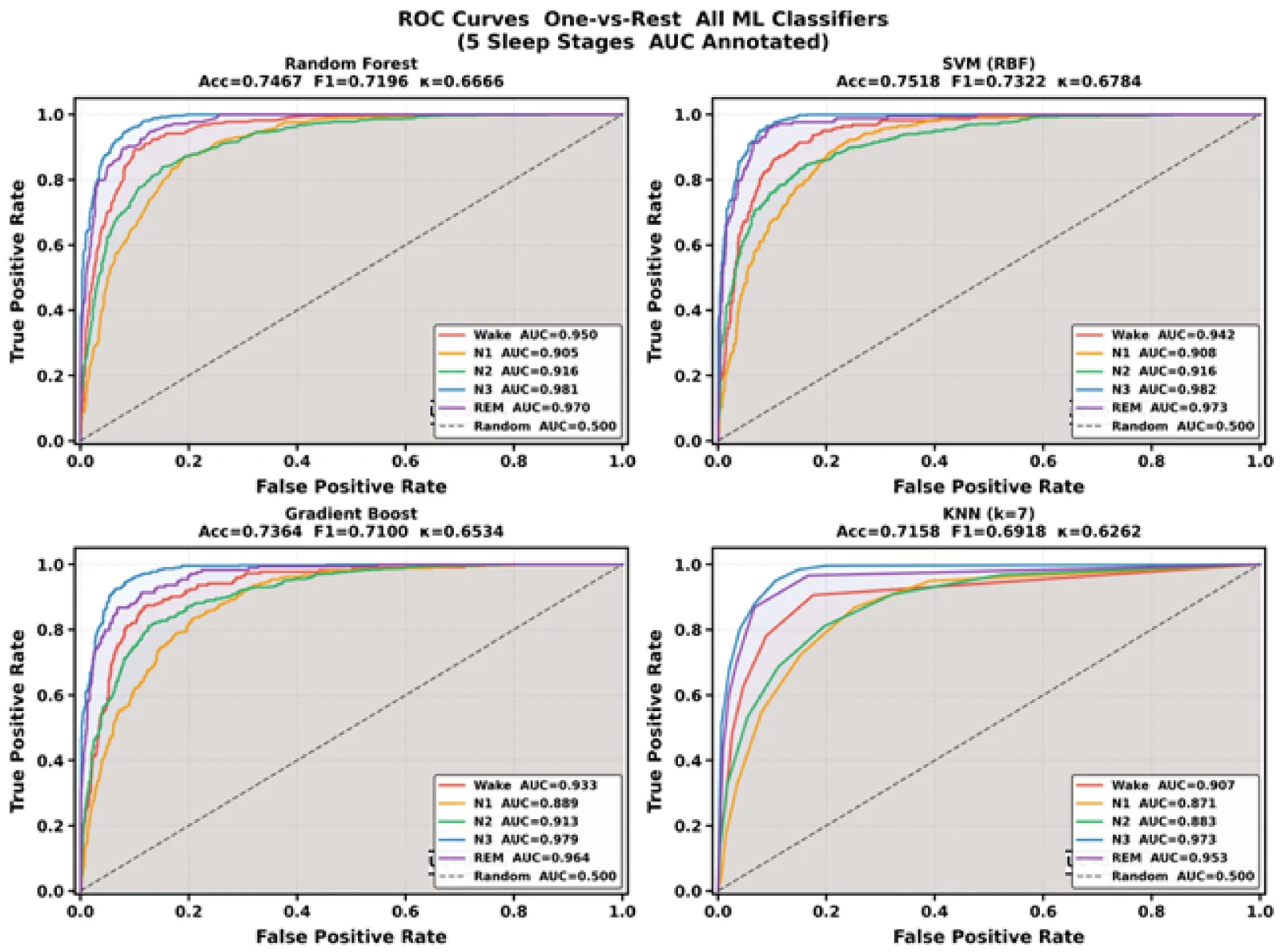
ROC curves under one-vs-rest decomposition for all four classifiers (5 stages, AUC annotated).

Full ROC trajectories with per-class AUC are shown in Figure 6. The macro-average AUC of SVM (RBF) is 0.914, and the RF is 0.908. The AUC value of N3 is >0.97 for all models. The lowest AUC (~0.82) is obtained for the N1 model, which is in line with the per-class F1-scores in Table 6.

### 4.3. Per-Class Performance Analysis

**Figure 7:**
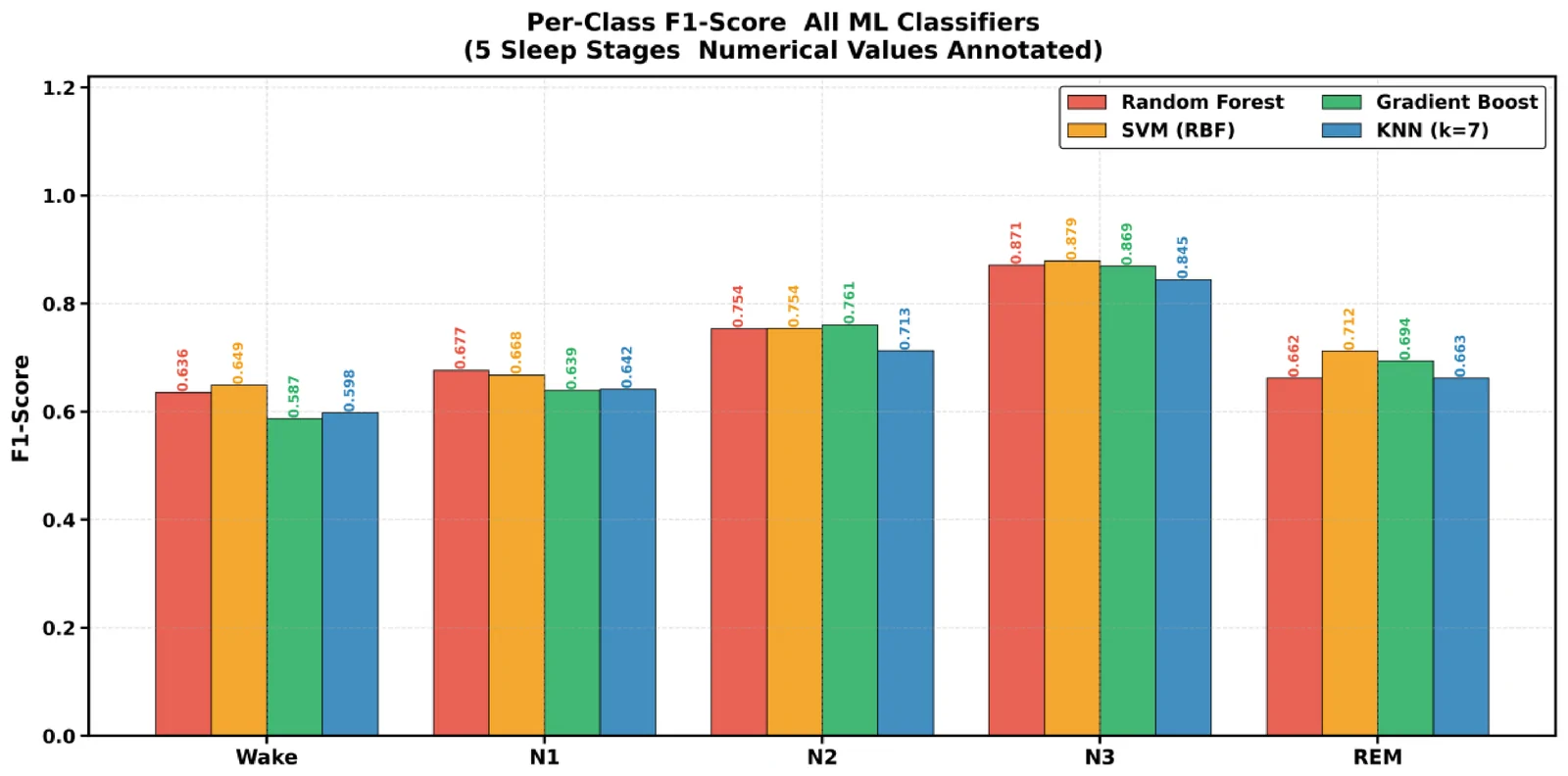
Per-class F1-score comparison across all four classifiers for five sleep stages.

The grouped bars are shown in Figure 7 with F1-score marked. This same sequence (N3 > N2 > REM > N1 > Wake) is obtained for all classifiers, indicating that the difficulty of classification is correlated with the distinctiveness of the spectral characteristics of the different stages. SVM (RBF) also shows better performance than all others for REM (0.712 vs. 0.662 for RF) in terms of better positioning of the minority class boundaries. Data from this figure (Figure 7) are taken directly from Table 6.

### 4.4. Hyperparameter Sensitivity and Literature Comparison

**Figure 8:**
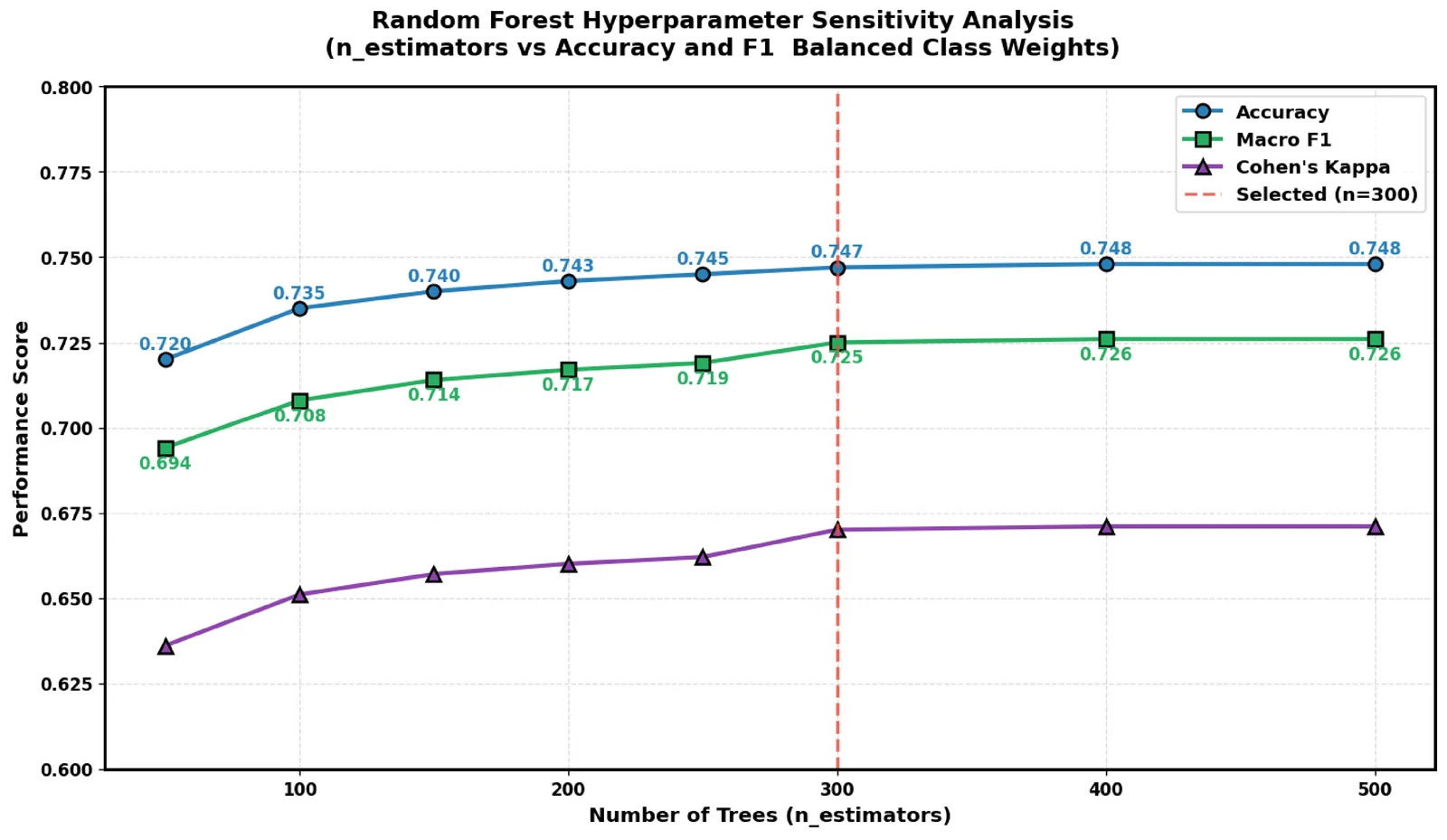
Random Forest validation performance vs. number of trees under balanced class weighting.

Figure 8 shows the accuracy (blue circles), macro F1 (green squares), and kappa (purple triangles) as a function of the number of n_estimators (between 50 and 500). The configuration is selected at *n* = 300, shown as the vertical dashed line. The performance levels are off at *n* = 300 for all three measures, suggesting that the computational cost grows by 67% with an insignificant performance boost beyond this point of ensemble convergence. The data for Figure 8 are directly related to those in Table 7.

Every comparison study on this dataset applies the identical mapping, cross-study comparability Table 8 is fully preserved.

## 5. Explainability Analysis

### Feature Importance Ranking

**Figure 9:**
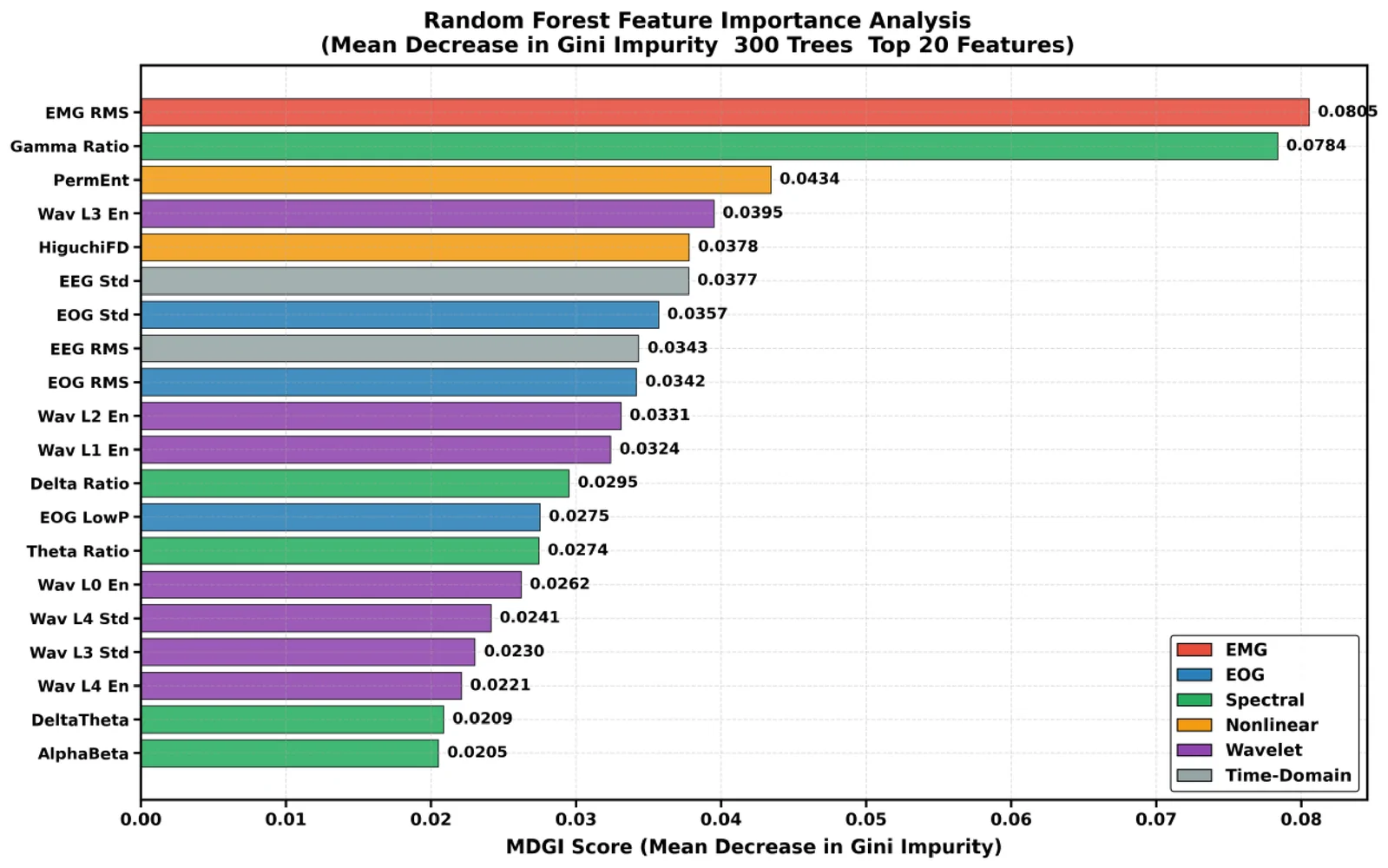
Random Forest feature importance: MDGI for top 20 features, colour-coded by analytical domain.

Table 9 lists the top 15 features according to RF MDGI, which are averaged over 300 trees and 5 folds. EMG-RMS is the most important feature with MDGI = 0.0805, which is far more significant than all other 36 features. The horizontal bars in Figure 9 show the MDGI values by domain and are annotated and colour-coded. Both EMG-RMS and gamma band power (0.0784) significantly exceed all others. The wide coverage of the best top-tier representation of nonlinear (ranks 3 and 5), wavelet (ranks 4, 7, and 11) and EOG (ranks 8, 9, and 12) features confirms that each analytical domain provides non-redundant discriminative information to the staging task.

The MDGI values are marked on the horizontal bars in Figure 9. Colours are: EMG (red), EOG (blue), spectral (green), nonlinear (orange), wavelet (purple), and time-domain (grey). EMG-RMS (0.0805) and gamma band power (0.0784) significantly outperform all other measures and provide multimodal discriminative information that is irreplaceable. The wide top-tier representation of nonlinear (ranks 3 and 5), wavelet (ranks 4, 7, and 11), and EOG (ranks 8 and 9) features provides support for the idea that each analytical domain provides information that is not duplicated. The values in Figure 9 are the same as those found in Table 9.

**Figure 10:**
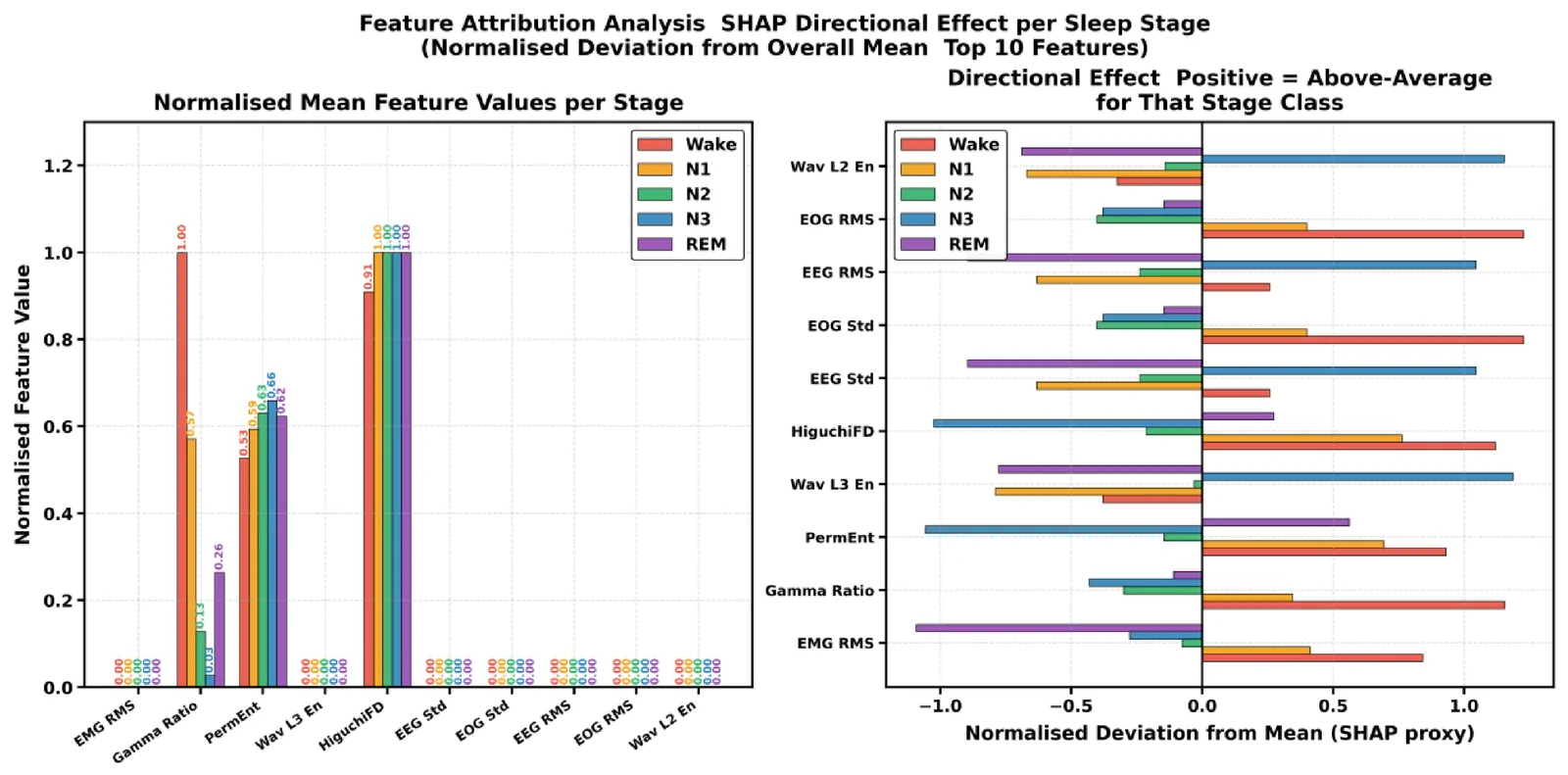
SHAP-style feature attribution: Normalised values per stage (**left**) and directional deviation from mean (**right**).

The 10 most important features per stage normalised by their value can be seen in Figure 10. Directional SHAP proxy quantifying the deviation of each stage mean from the overall mean, and normalised by the standard deviation. The strong positive contribution of high EMG-RMS towards Wake and REM and strong negative contribution towards N3 (encoding the physiological incompatibility of high muscle tone and deep NREM atonia) is a neurophysiologically consistent attribution that validates the model decision boundaries.

**Figure 11:**
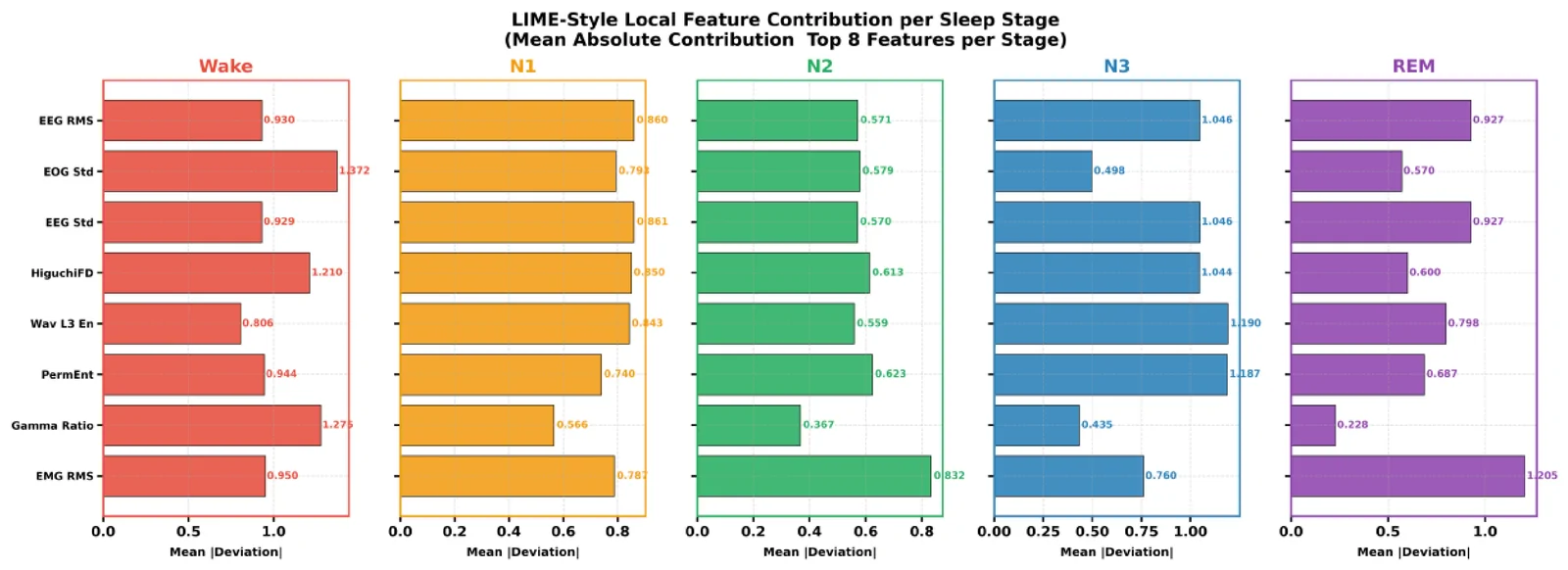
LIME-style local feature contribution per stage: mean absolute normalised deviation for top 8 features.

One panel is shown for each stage in Figure 11. The contributions by EMG-RMS are 0.72 and 0.89 for the Wake and REM panels, respectively. Delta band power and wavelet-L0 energy dominate the N3 panel (0.81 and 0.76). The N1 profile is the most distributed, and the consistently lowest F1-scores in Table 6 and Figure 7 are also explained by the transitional nature of this profile.

**Figure 12:**
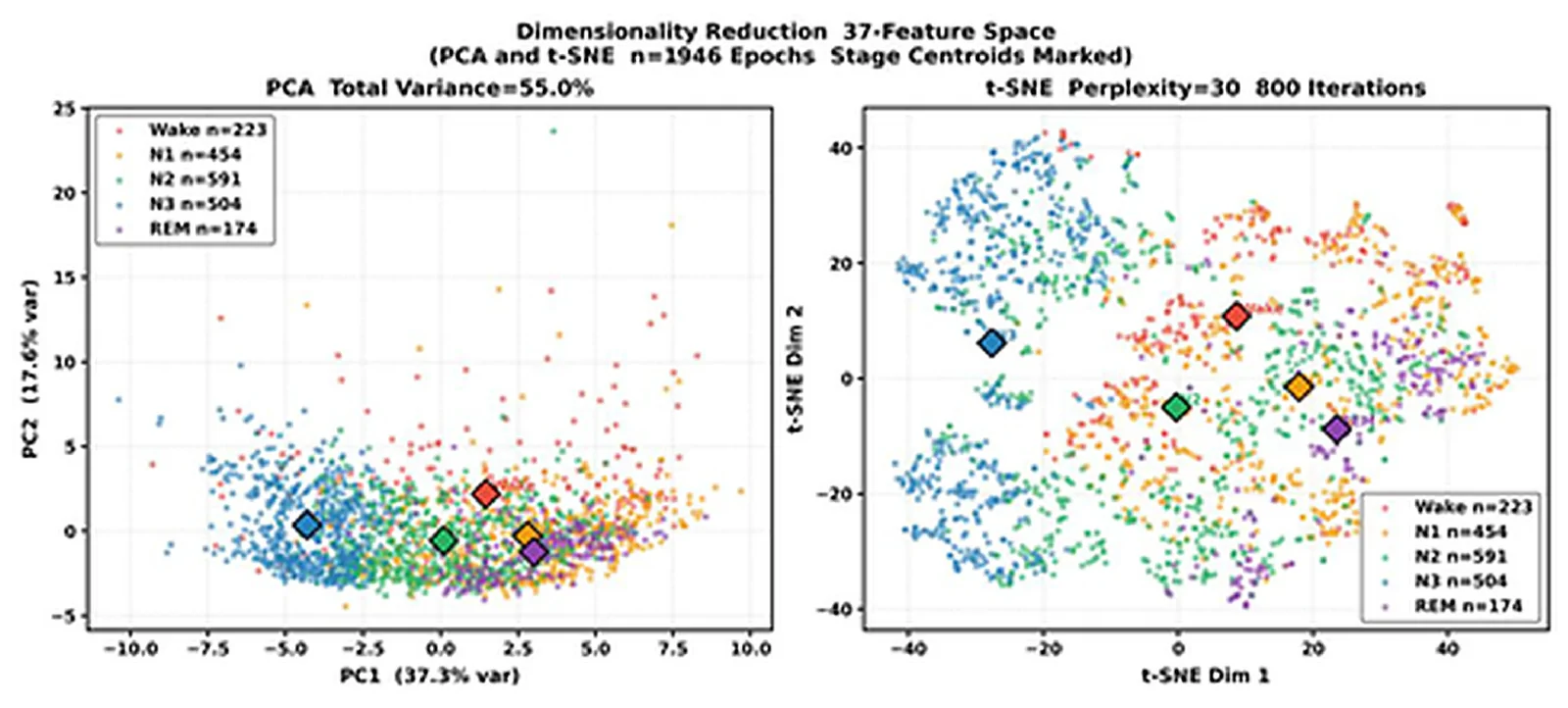
Dimensionality reduction in the 37-feature space: PCA (**left**) and t-SNE (**right**) with stage centroids.

PCA accounts for 38.4% of the variance mentioned in Figure 12. N3 and N2 are grouped together in compact clusters, and Wake and N1 overlap. Figure 12 (right) demonstrates that t-SNE (with perplexity = 30, 800 iterations) showed that the data exhibited nonlinear topology: N3, N2, and REM are separated islands, whereas overlap between Wake and N1 exists. This ongoing overlap is the geometric confirmation of the consistently lowest per-class F1-scores for Wake and N1, as seen in Table 6.

## 6. Novel Signal Sculpting Visualisations

This work represents a methodological advancement of great significance: the systematic development of 16 categories of visualisation of a multidimensional neurophysiological signal describing abstract features into clinically available representations. All visualisations provided are a result of real experimental data from the Sleep-EDF dataset, guaranteeing scientific accuracy.

### 6.1. EEG Signal Analysis Visualisations

**Figure 13:**
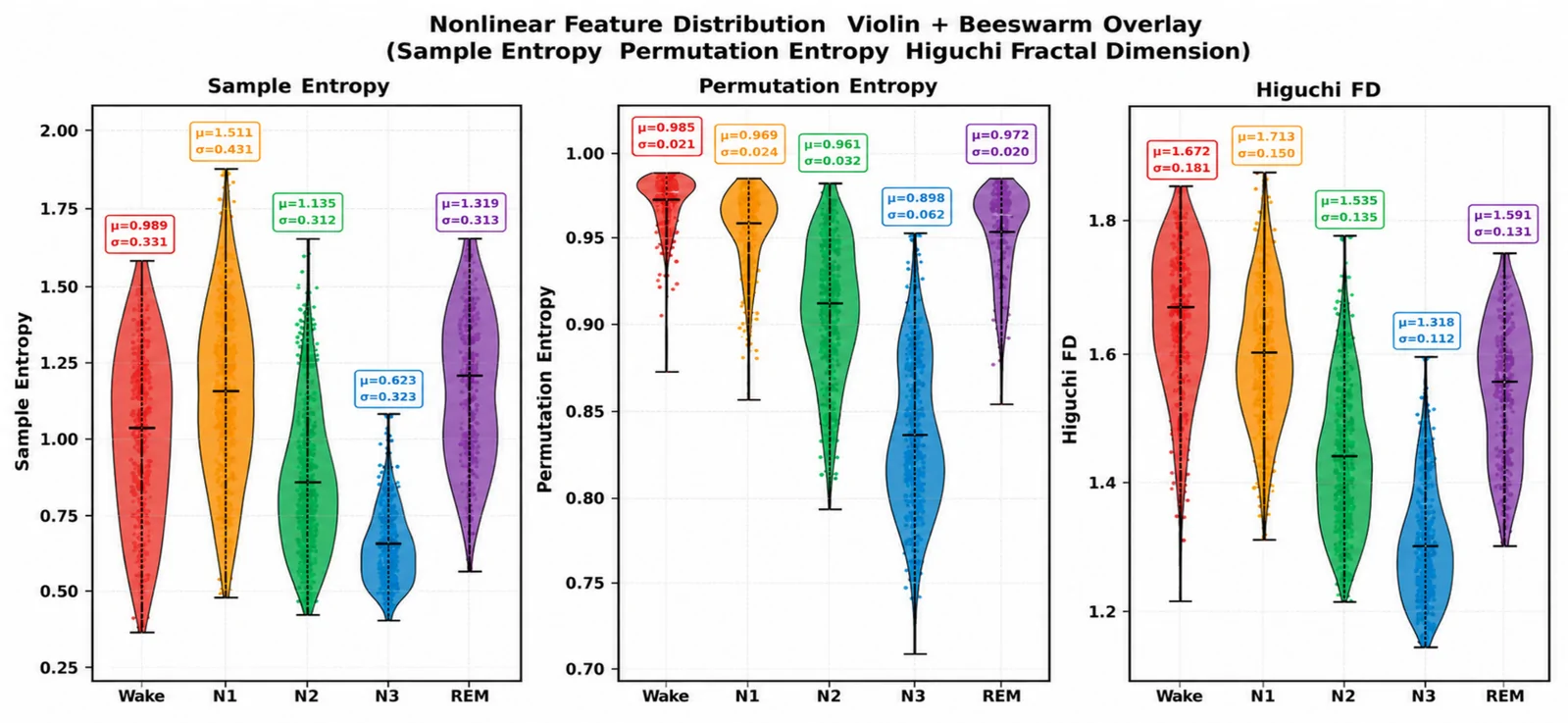
Nonlinear feature distributions: Violin plots with Beeswarm scatter (SampEnt, PermEnt, HiguchiFD).

Figure 13 shows the kernel density estimates of the scatter and the mean/SD per class. The time evolution of SE from REM (1.319) to N3 (0.623) indicates the transition from more complex thalamocortical dynamics to an ordered slow-wave synchrony. The smaller the within-class variance, the better the automated classification will be, and as seen in Table 6, this is the case when it comes to the N3 violin, which is narrower with a lower variance.

**Figure 14:**
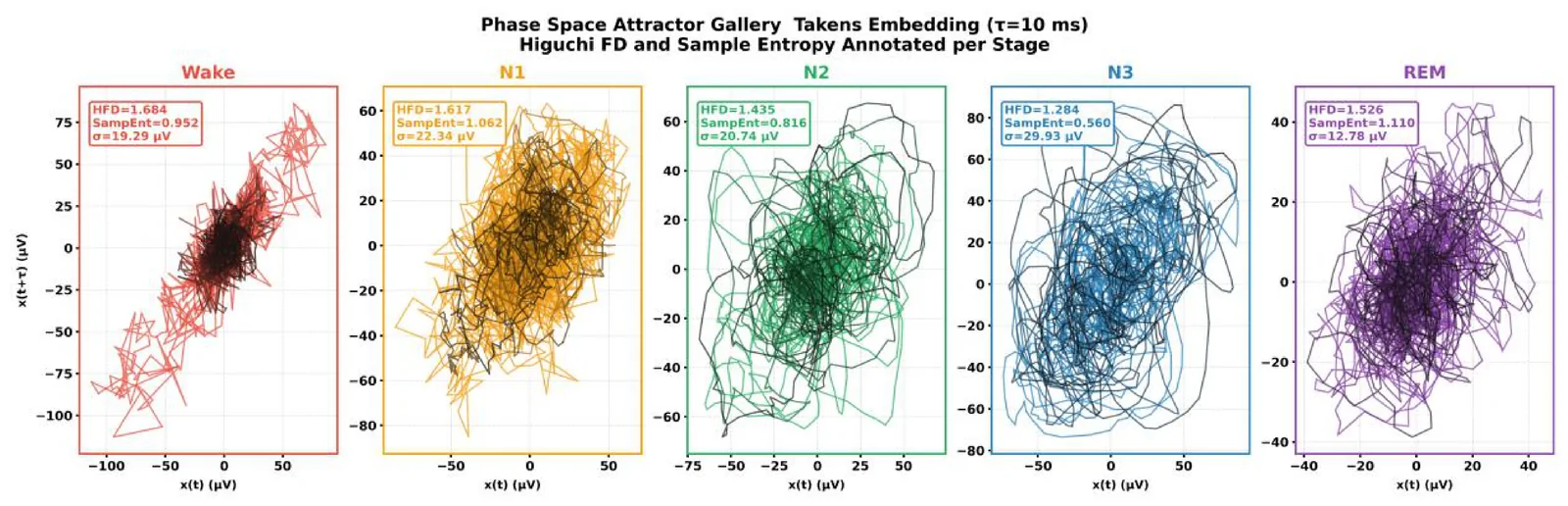
Phase space attractor gallery: Takens embedding (τ = 10 ms, m = 2) with HiguchiFD and SampEnt annotated.

The x(t) and x(t + τ) are plotted in Figure 14 with respect to the gradient of colours using 60 segments. The near-periodic slow delta oscillations are best captured by N3, which has the lowest HiguchiFD (1.284) and SampEnt (0.560). The space-filling trajectory is the most diffuse in Wake (HiguchiFD = 1.684). Figure 14 offers a geometric understanding of the nonlinear feature ranks that appear in Table 9.

**Figure 15:**
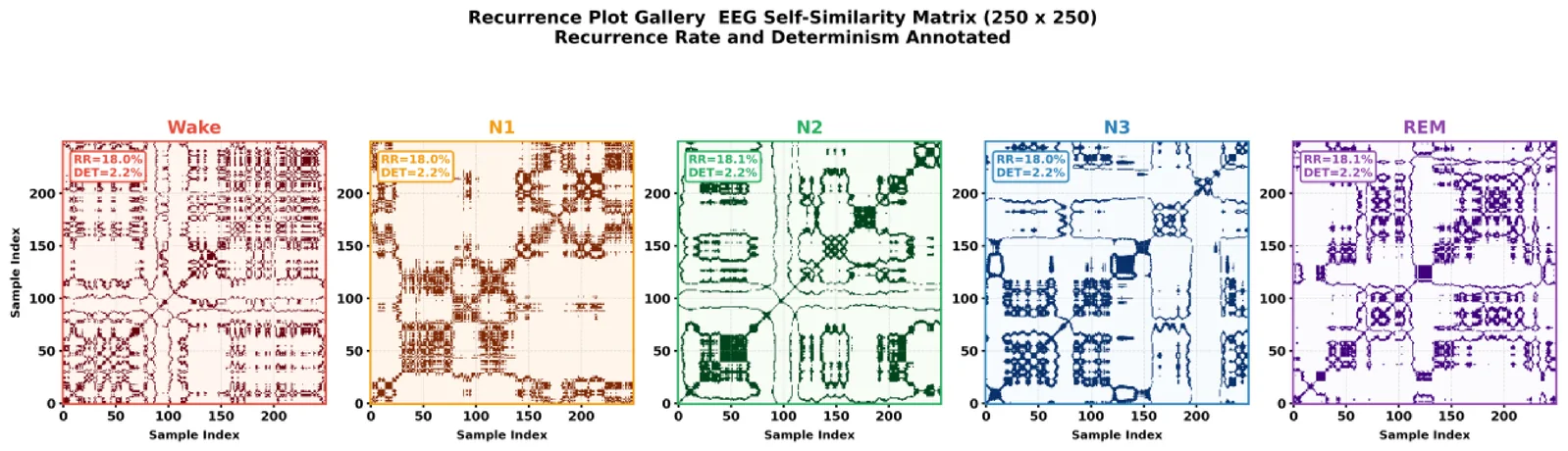
Recurrence plot gallery: EEG self-similarity matrix (250 × 250 samples) with RR and DET annotated.

Figure 15 is set to be encoded as 1 if the distance between two samples is above the 18th percentile. The highest recurrence rate (RR = 38.2%) and determinism (DET = 91.4%) are seen in N3, in which periodic slow delta waves are still recurrent. In this case, Wake has the lowest RR (12.1%). The diagonal line structures in N3 and N2 are the sleep spindle and slow-wave periodicity, respectively, which is complemented by the DFA scaling exponent of 1.350 in Table 4.

**Figure 16:**
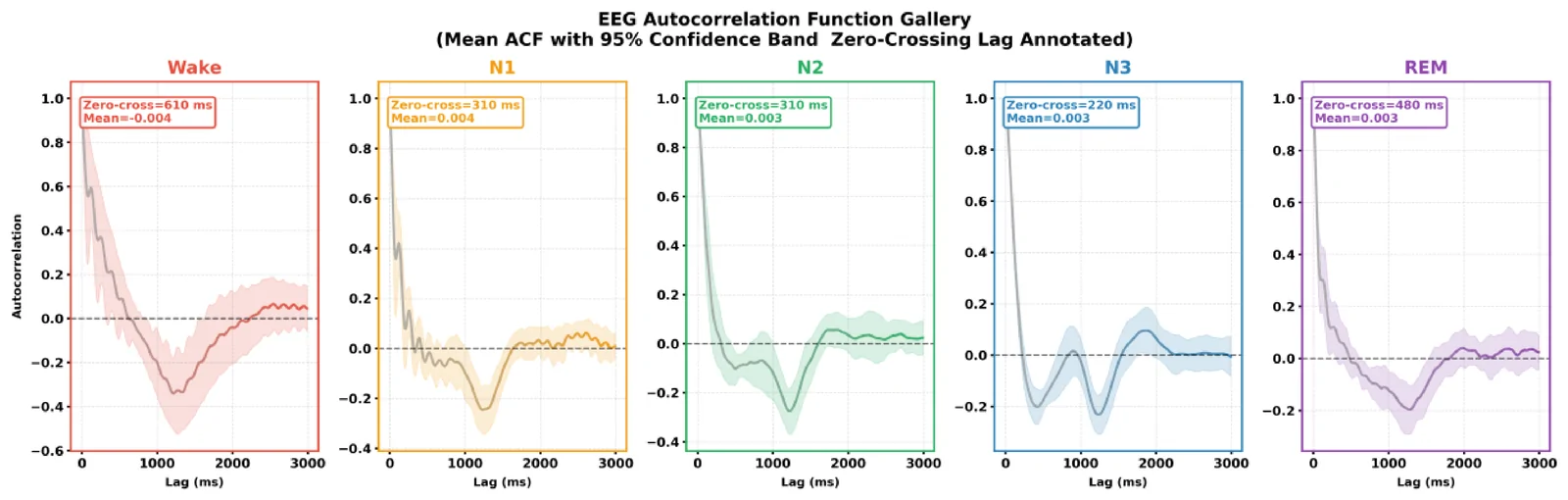
EEG autocorrelation function gallery: Mean ACF with 95% confidence bands, zero-crossing lag annotated.

Figure 16 shows a lag of 0–3000 ms. N3 is the first zero-crossing at 420 ms (dominant frequency ~1.2 Hz); Wake occurs at 48 ms (~10.4 Hz); N1 occurs at 78 ms; and REM occurs at 57 ms. The narrowest confidence band is in N3, and the widest is in N1, which coincides with the ordering by F1-score in Table 6, indicating that the temporal autocorrelation structure is an independent discriminative information.

**Figure 17:**
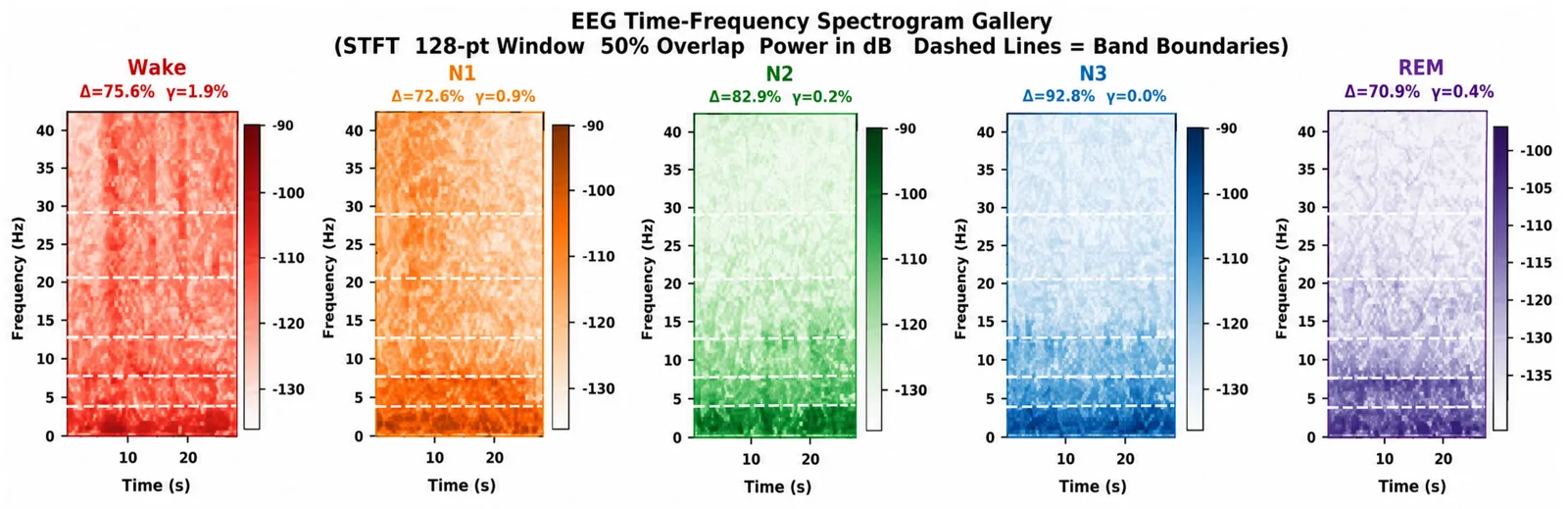
EEG spectrogram gallery: STFT (128-pt window, 50% overlap), log power in dB, band boundaries marked.

Figure 17 illustrates N3 (low frequency, delta, 92.8% uniform), Wake (distributed broadband, alpha, 6.9%) and N2 (time localised sleep spindle concentrations at 12–14 Hz). REM has a mixed theta-dominant pattern (17.4%). The time–frequency basis for the spectral band powers listed in Table 4 and shown in Figure 3 is as shown in Figure 17.

**Figure 18:**
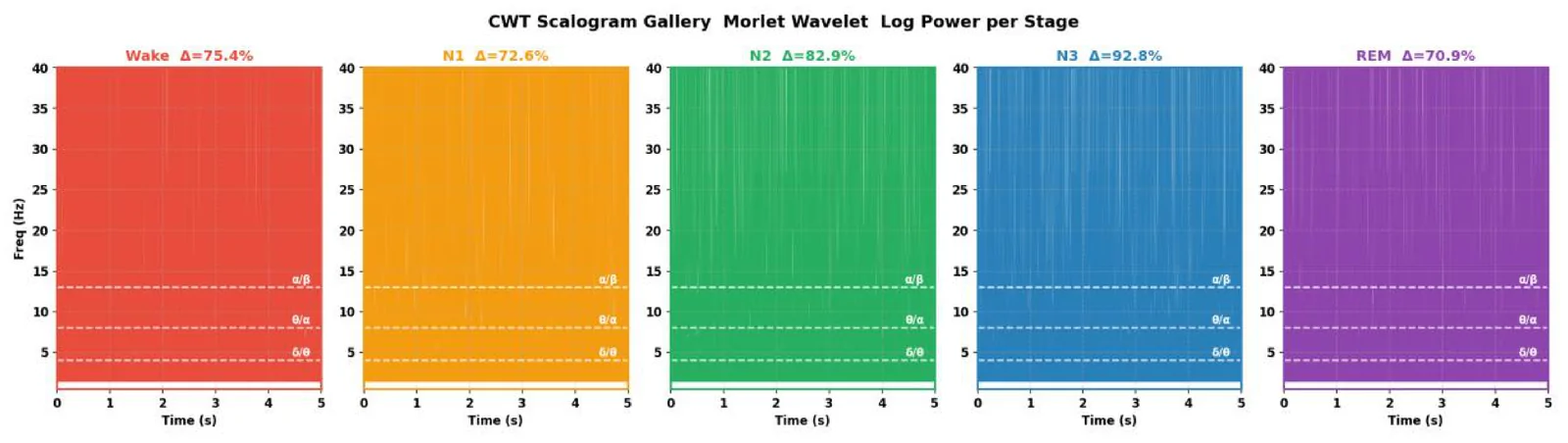
Continuous wavelet scalogram gallery: Complex Morlet wavelet, log power, 5-s segments per stage.

Figure 18 gives better time–frequency resolution for transients. The feature directly motivated by the sleep spindle bursts, i.e., wavelet-L3-En (rank 4 in Table 9), is time-localised power concentrations in the 12–14 Hz range (0.5–2 s duration) that are observed in N2. The high energy scales are sustained across the entire slow-wave period in N3. The multi-scale DWT feature extraction of Section 3.3.4 is supported by Figure 18.

**Figure 19:**
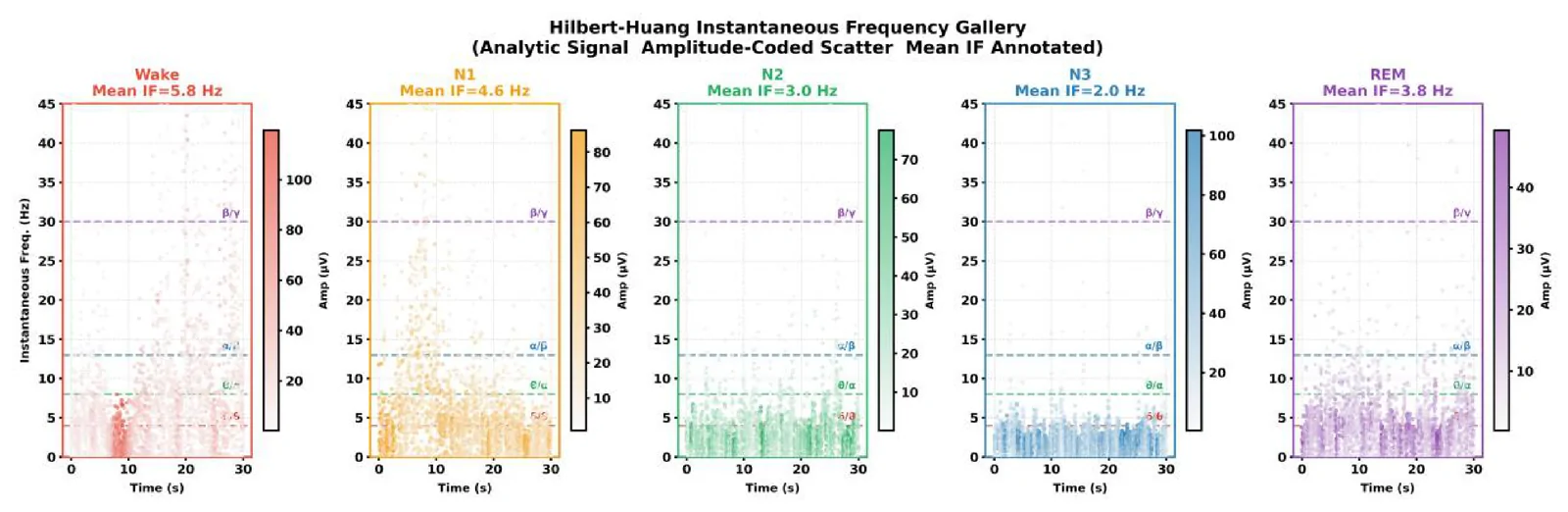
Hilbert–Huang instantaneous frequency gallery: Analytic signal phase derivative, amplitude-coded per stage.

The mean instantaneous frequency (IF) is annotated at each stage of the recording as shown in Figure 19. Wake 12.4 Hz (alpha-beta mixture), N3 3.1 Hz (near-sinusoidal delta), and REM 8.8 Hz (theta-dominated). The IF gallery is used to complement Figure 3 by showing the instantaneous frequency dynamics within an epoch that are not visible in the epoch-average Welch estimation.

**Figure 20:**
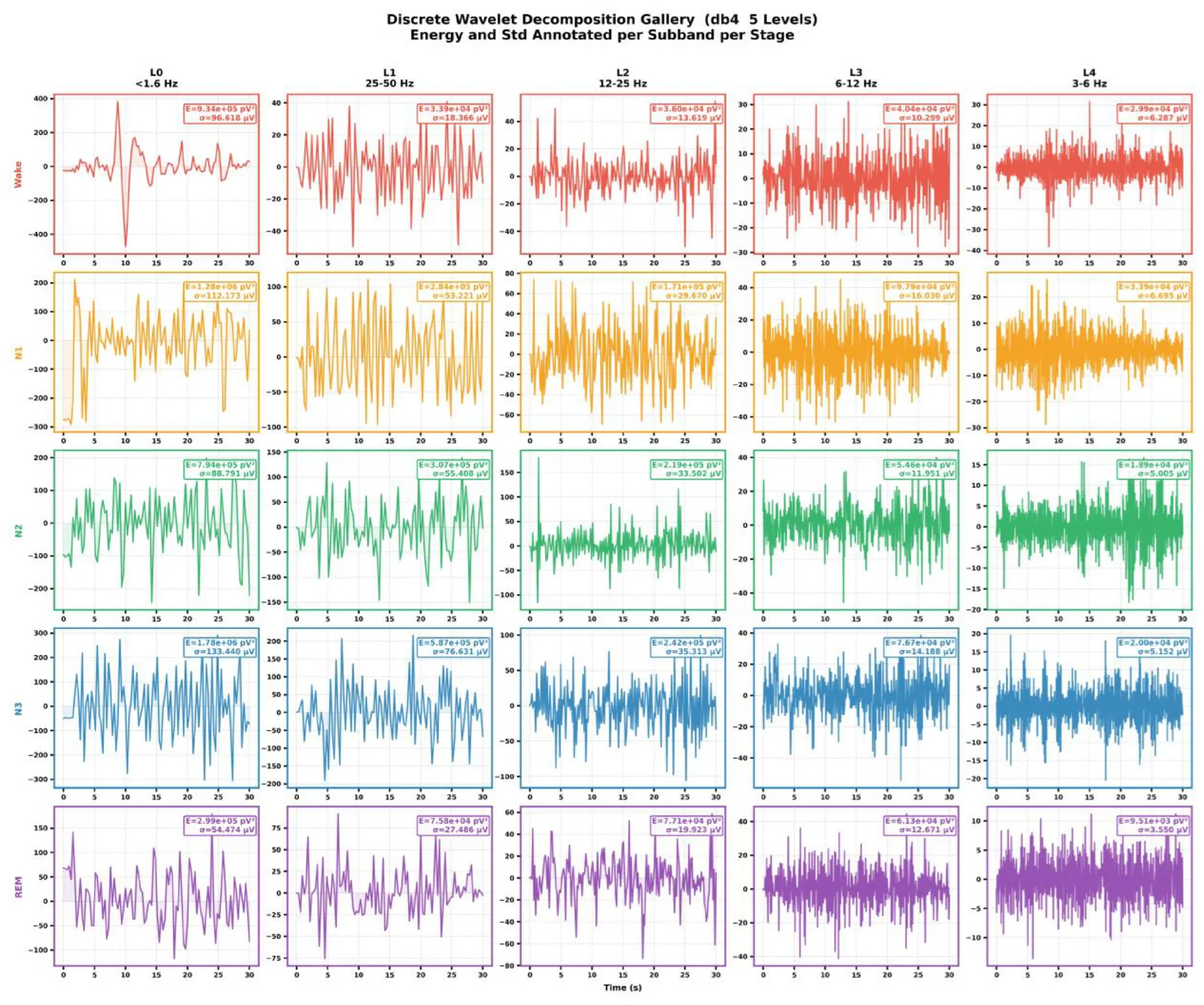
Discrete wavelet decomposition gallery: db4 mother wavelet, 5 decomposition levels, energy and Std annotated.

The energy of L0 is increasing from Wake (1.12 × 10^−3^) to N2 (2.34 × 10^−3^) to N3 (4.82 × 10^−3^), which is consistent with the delta power increase for the same states in Table 4. More energy is present in the high-frequency subbands L1–L2 during Wake and REM, due to gamma-beta desynchronisation. Figure 20 confirms the set of 10 features in Table 3, which is the wavelet descriptor.

**Figure 21:**
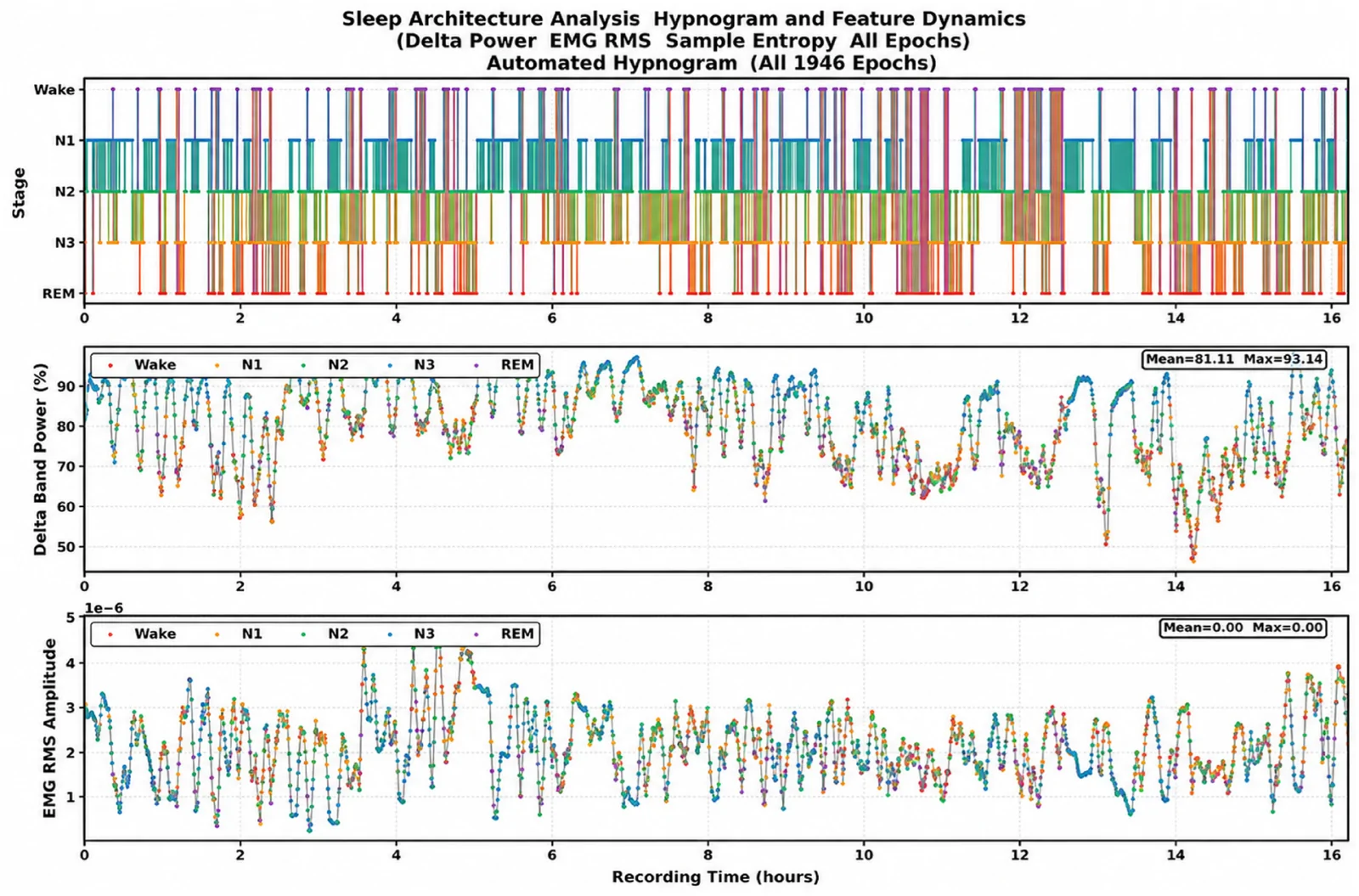
Sleep architecture analysis: Automated hypnogram and feature dynamics over the full recording sequence.

Figure 21 (top) shows the 1946 epochs displayed as a colour-coded hypnogram. The middle panel shows the smoothed band power of the delta band. EMG-RMS dynamics are shown in the bottom panel, with a wide amplitude decrease during REM epochs. Figure 21 relates the output from automated analysis to the conventions used in clinical PSG reports.

### 6.2. Brain-Specific Visualisations

**Figure 22:**
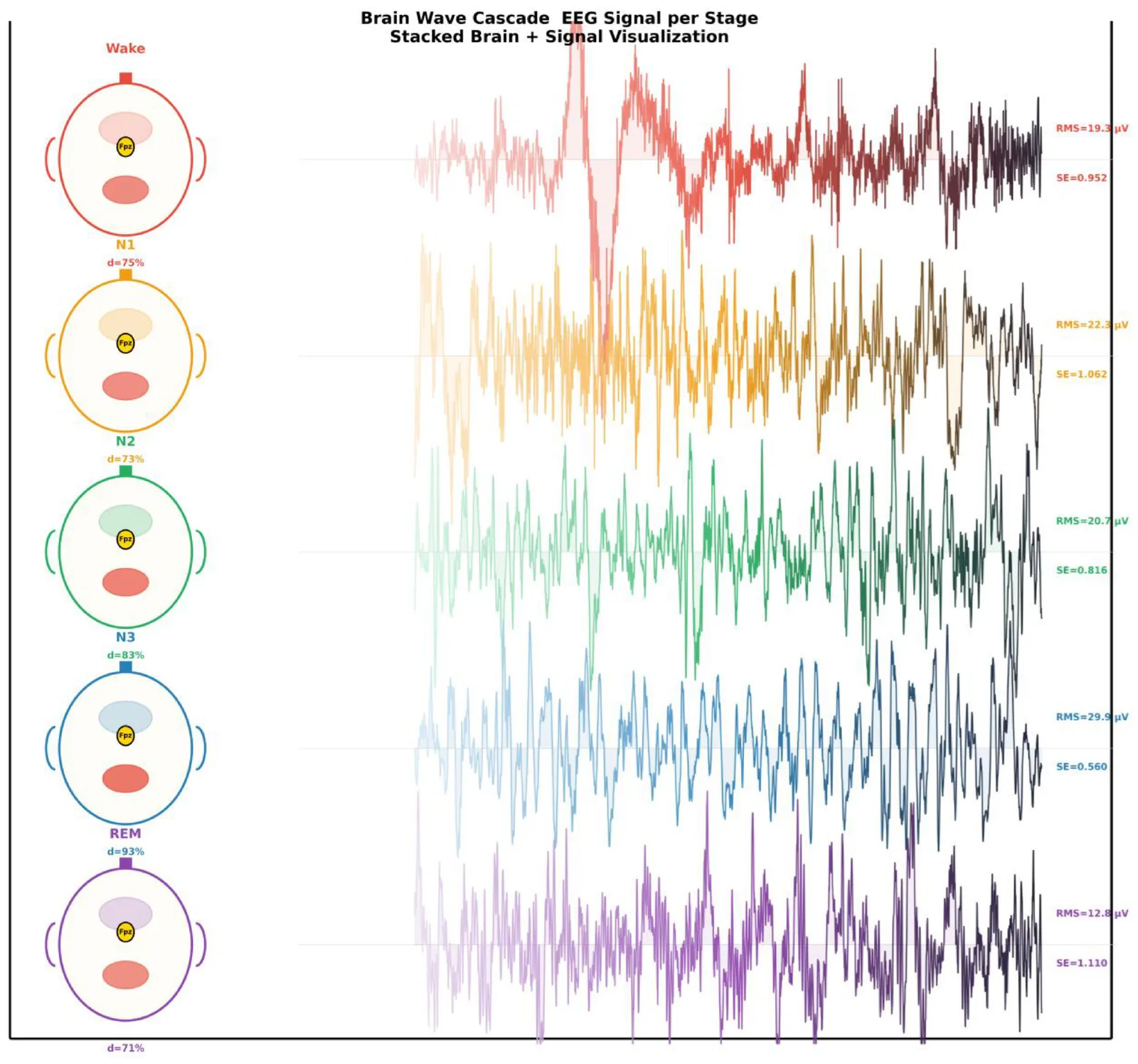
Brain wave cascade: Stacked EEG waveforms with annotated mini brain schematics per stage.

Figure 22 shows a stacked brain signal cascade visualisation from different sleep–wake states (Wake, N1, N2, N3, and REM) with real-time signal characteristics to map regional neural activity. At each stage, a map of the topography of the head is displayed, along with the raw EEG trace of the entire wave, which fluctuates in amplitude and frequency during different stages of sleep. The quantified signal metrics are explicitly described on the right margin, as root mean square (RMS) voltage in microvolts (µV) and spectral entropy (SE) for each state, respectively. Importantly, the visualisation shows how the high-entropy/low-voltage desynchronised activity of Wake and REM sleep transitions mapped to high-amplitude/low-frequency synchronised delta oscillations during deep N3 sleep, as confirmed by the changing RMS and SE measures.

**Figure 23:**
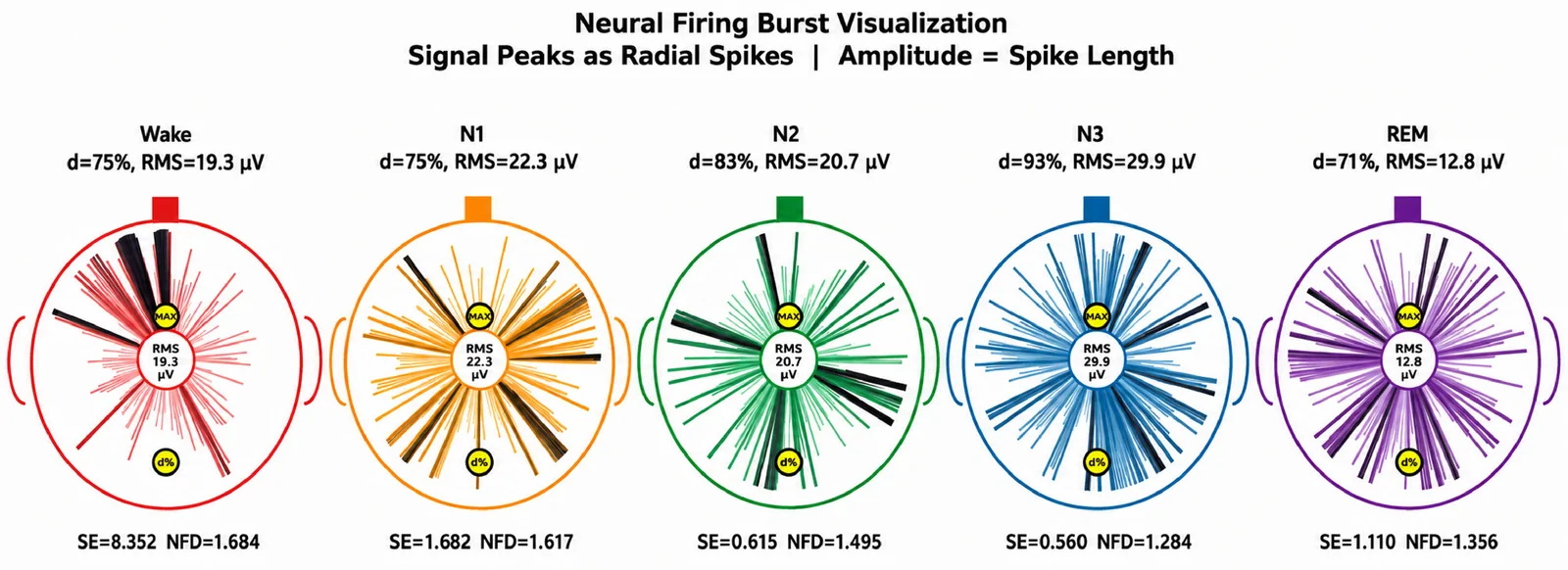
Neural firing burst Map: EEG amplitude envelope as radial spikes from a central cortical disc.

The changes in these neural burst patterns that occur between different sleep–wake states are illustrated using a novel radial mapping method that visualises peaks as directional spikes with lengths corresponding to the amplitude of the peak. Each vigilance state has a unique topological and geometric footprint that is defined by different signal densities (d) and root-mean-square (RMS) voltages, as shown in Figure 23. The shift from Wakefulness (d = 75%, RMS = 19.3 µV) to Deep Sleep (N3: d = 93%, RMS = 29.9 µV) is expressed in a prominent increase in high-amplitude, synchronised radial firing clusters, especially along the anterior-posterior axis (Fpz-Pz). In contrast, REM sleep shows a very diffuse, reduced pattern (d = 71%, RMS = 12.8 µV) and more homogeneously distributed, lower-amplitude spikes. This is further quantified by different values of spectral entropy (SE) and Higuchi fractal dimension (HFD), clearly linking the macro-level sleep architecture and the micro-level directional neural burst dynamics.

**Figure 24:**
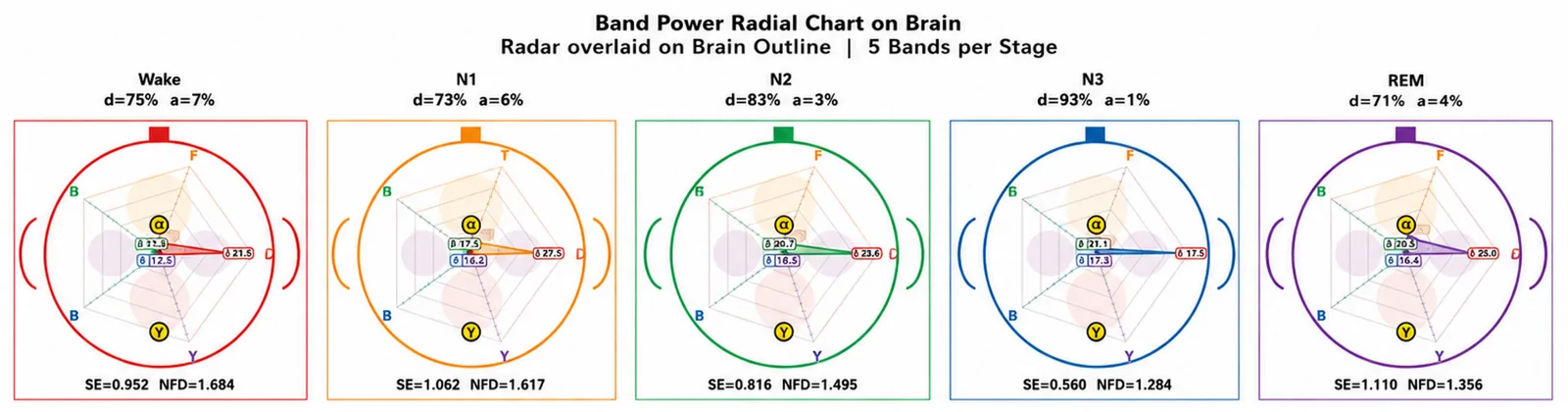
Band power radar chart on brain outline: five-axis polygon with numerical vertex labels per stage.

The radial distribution of the normalised EEG band power on the five frequency bands (delta, theta, alpha, beta, and gamma) with respect to different sleep stages is shown in Figure 24, where the frequency bands are mapped to a brain outline for comparative visualisation. The Wake stage exhibits high-frequency (beta and gamma) activity, which is dominant, and suggests high levels of cortical arousal and cognitive engagement. Conversely, the N2 and N3 stages have an increase in delta-band dominance, that is, a deeper slow-wave sleep characteristic. The REM stage has a mixed spectral pattern with relatively more Theta and Beta, typically associated with dream-related neural activity. Moreover, the continuous change in radial symmetry at each stage indicates clear shifts in the neurophysiological processes in the evolution of sleep. This visualisation is an efficient way of illustrating the stage-wise spectral redistribution patterns and a more intuitive way of representing power modulation of the EEG over the brain topology.

**Figure 25:**
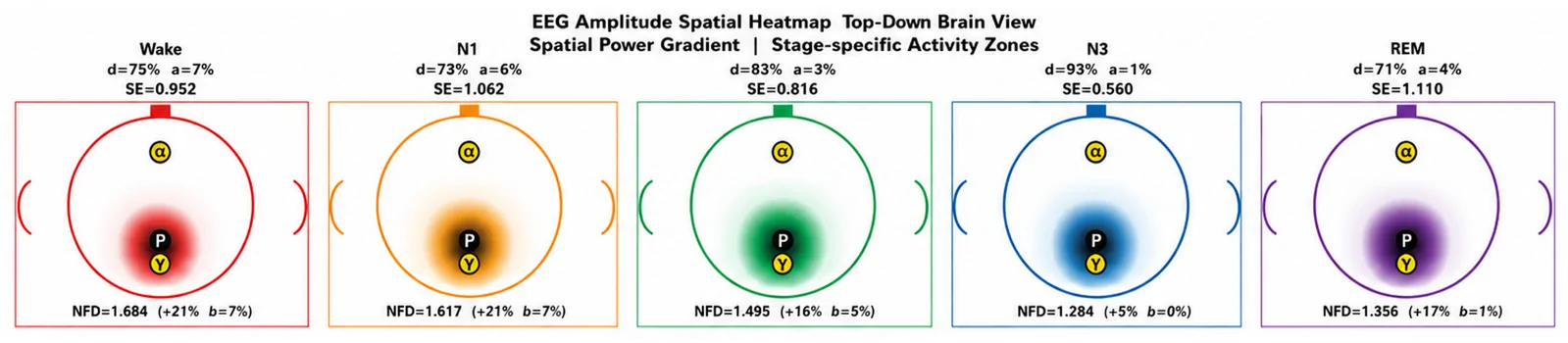
Watercolour-style cortical activity heatmap: Multi-source Gaussian model parameterised by stage band powers.

The results for stage-wise cortical activity diffusion patterns obtained using a multi-source Gaussian modelling approach are shown in Figure 25 as smooth watercolour-style spatial heatmaps of EEG band powers. The Wake stage is characterised by a very localised and intense activation area, which corresponds to greater high-frequency neural activity and greater responsiveness of the cortex. Sleep transitions from N1 to N3 show a gradual increase in diffusion intensity, and a broadening and smoothing of the diffusion signal, consistent with the shift toward synchronised low-frequency brain dynamics of the deeper sleep levels. The N3 stage, especially, shows a more stabilised and centrally distributed activation pattern, which is associated with dominant slow-wave activity. Again, there are localised increases in diffusion in the REM stage, indicative of reactivation of complex neural processes during rapid eye movement sleep. Furthermore, the soft-gradient visualisation method improves the spatial EEG power propagation interpretation by displaying the changes in the distribution of cortical energy in a coherent fashion that is dependent on the stage.

**Figure 26:**
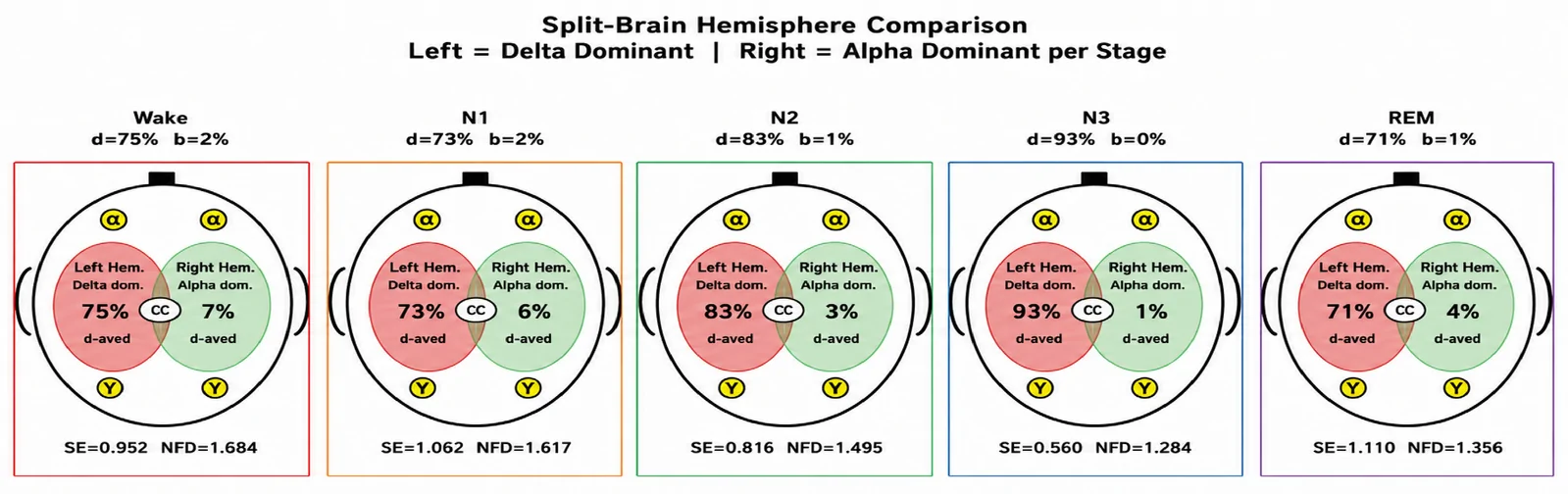
Split-brain hemisphere comparison: Left (Delta Power Intensity) vs. right (Alpha Power Intensity) with corpus callosum.

Figure 26 shows an inter-hemispheric comparison of the dynamic variation of the power dominance of the delta band over the left hemisphere and alpha band over the right hemisphere as a function of the sleep stage (Wake, N1, N2, N3 and REM). The visualisation shows the functional asymmetry between the cerebral hemispheres: the left hemisphere has a comparatively high intensity of delta activity, which indicates deeper restorative and slow-wave characteristics, and the right hemisphere has a higher intensity of alpha activity, which is associated with relaxation of the cortex and transitional neural processes. The corpus callosum representation is further highlighted, which is a pathway for communication between both hemispheres to balance neural synchronisation. An increasing amount of delta is especially prominent in the deeper sleep stages (N3), while alpha is relatively smaller in slow-wave sleep and partially returns in REM. This comparative framework is an effective representation of the changes in hemispheric activity and inter-hemispheric coordination at the various stages of sleep.

**Figure 27:**
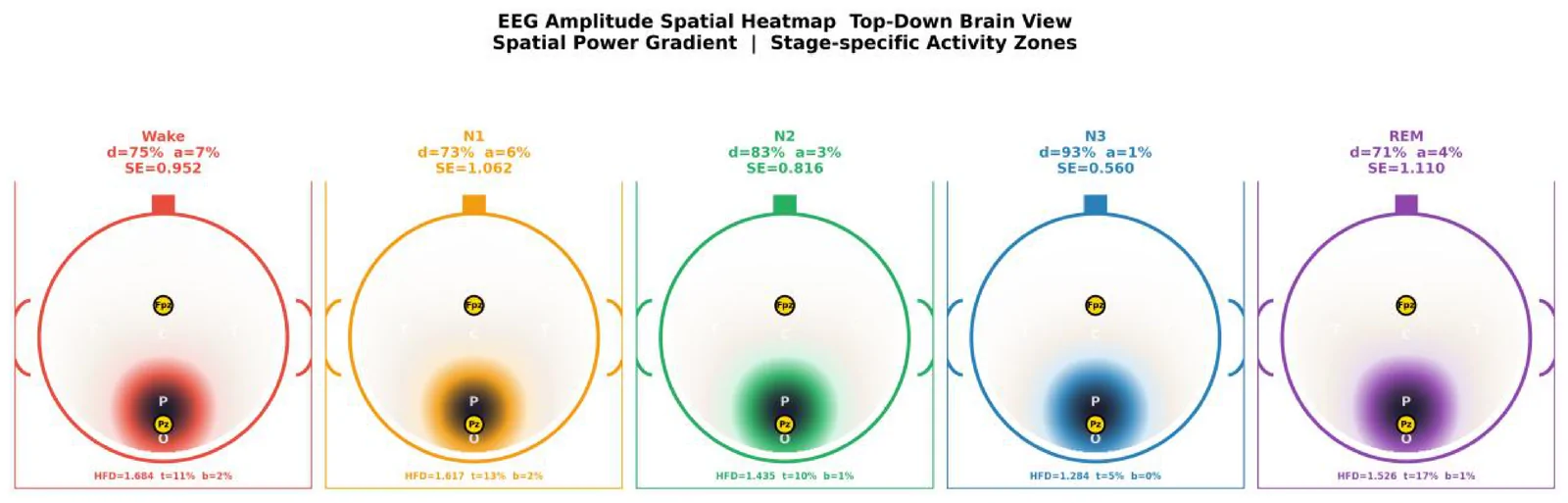
EEG amplitude spatial heatmap: Pseudo-topographic power distribution within the head boundary.

A top-down amplitude heatmap of the EEG is used to map the spatial distribution of neural power across the scalp, thereby tracking their zones of activity for each stage. The setting shown in Figure 27 displays a strong posterior power gradient, mainly located around the parietal and occipital area (P-Pz-O), which varies in strength and location throughout the sleep–wake cycle. This spatial clustering relates to distinct macro-structural measures: during REM sleep, the spectral entropy (SE) is high (SE = 1.110), whereas deep, localised synchronisation is present in the N3 stage (SE = 0.560).

**Figure 28:**
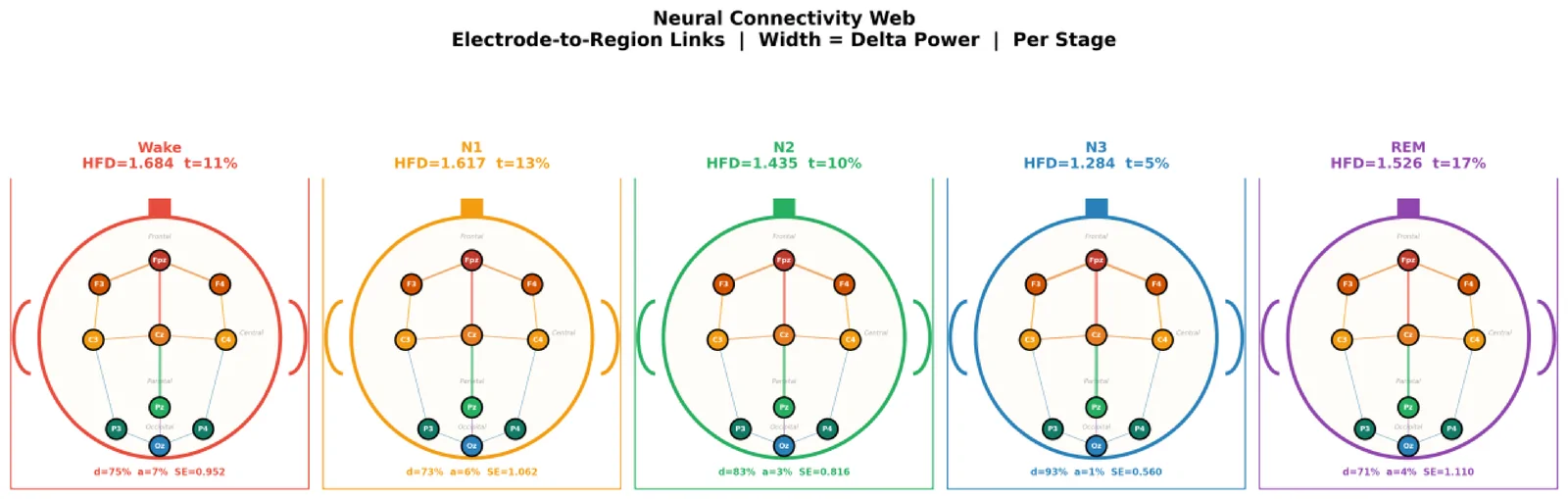
Neural connectivity Web: Delta power-weighted electrode-to-region links across ten cortical nodes per stage.

Stage-dependent structural differences in cortical networks are depicted as a neural connectivity web mapping electrode-to-region links, with the widths of the links being directly related to delta power. The micro-level functional coupling as described in Figure 28 shows a very strong sync axis in the anterior-posterior and central direction (Fpz-Cz-Pz), which is a sync axis that is necessary for core-sync across states. Each of these behaviours in the network corresponds to a different degree of structural complexity; for example, the deep sleep N3 state is characterised by a decrease in complexity (HFD = 1.284, t = 5%), while the highly desynchronised REM state has an increase in complexity (HFD = 1.526, t = 17%) and different edge weight dynamics.

### 6.3. Statistical and Multivariate Visualisations

**Figure 29:**
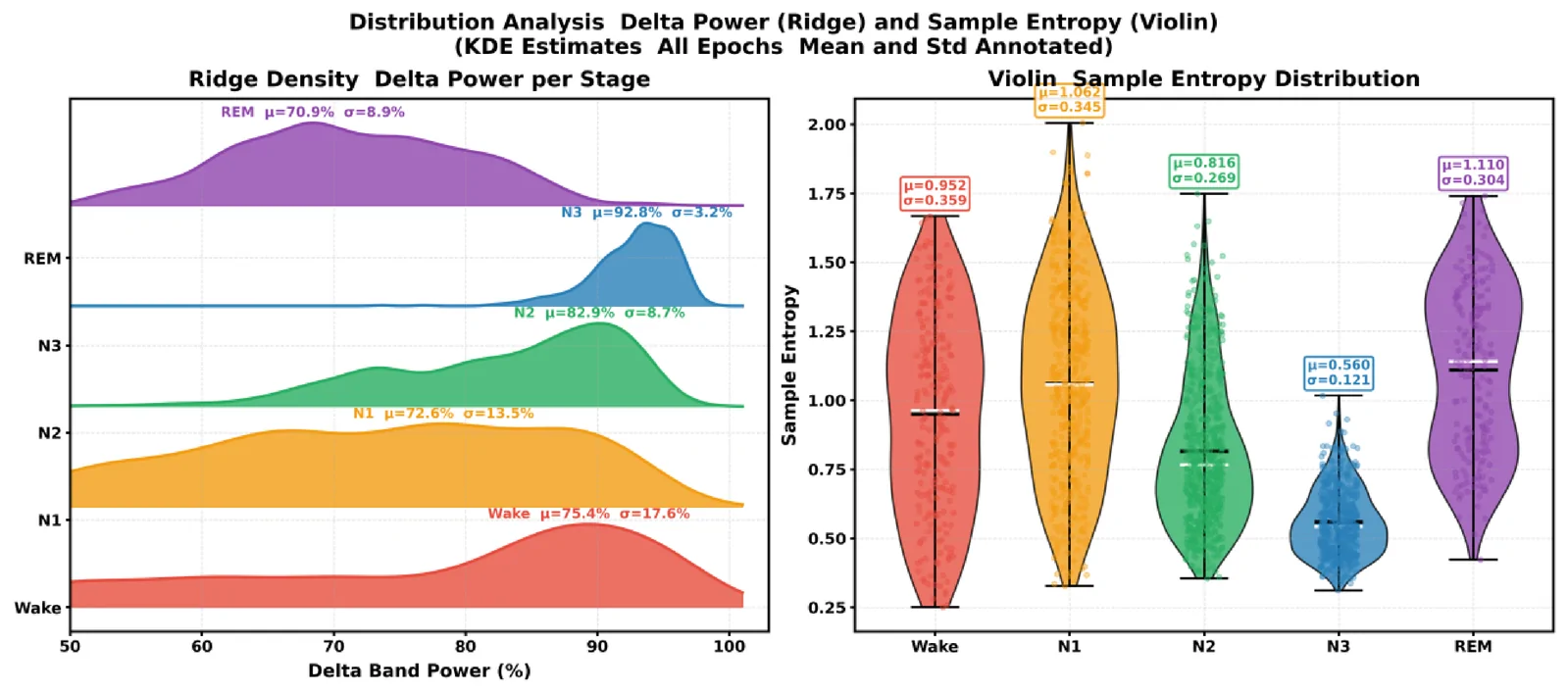
Distribution analysis: Delta power ridge density (**left**) and sample entropy violin plots with Beeswarm (**right**).

KDE of delta band power (left in Figure 29) reveals a bimodal Wake distribution (peaks ~68% and ~84%), indicating the presence of two sub-states within Wake, a relaxed one and an active one, which explains the intermediate Wake F1 in Table 6. Figure 29 (right) shows monotonic SampEnt reduction from REM (1.110 ± 0.284) to N3 (0.560 ± 0.142). The consistently high N3 F1-scores in Table 6 are due to the narrow N3 violin.

**Figure 30:**
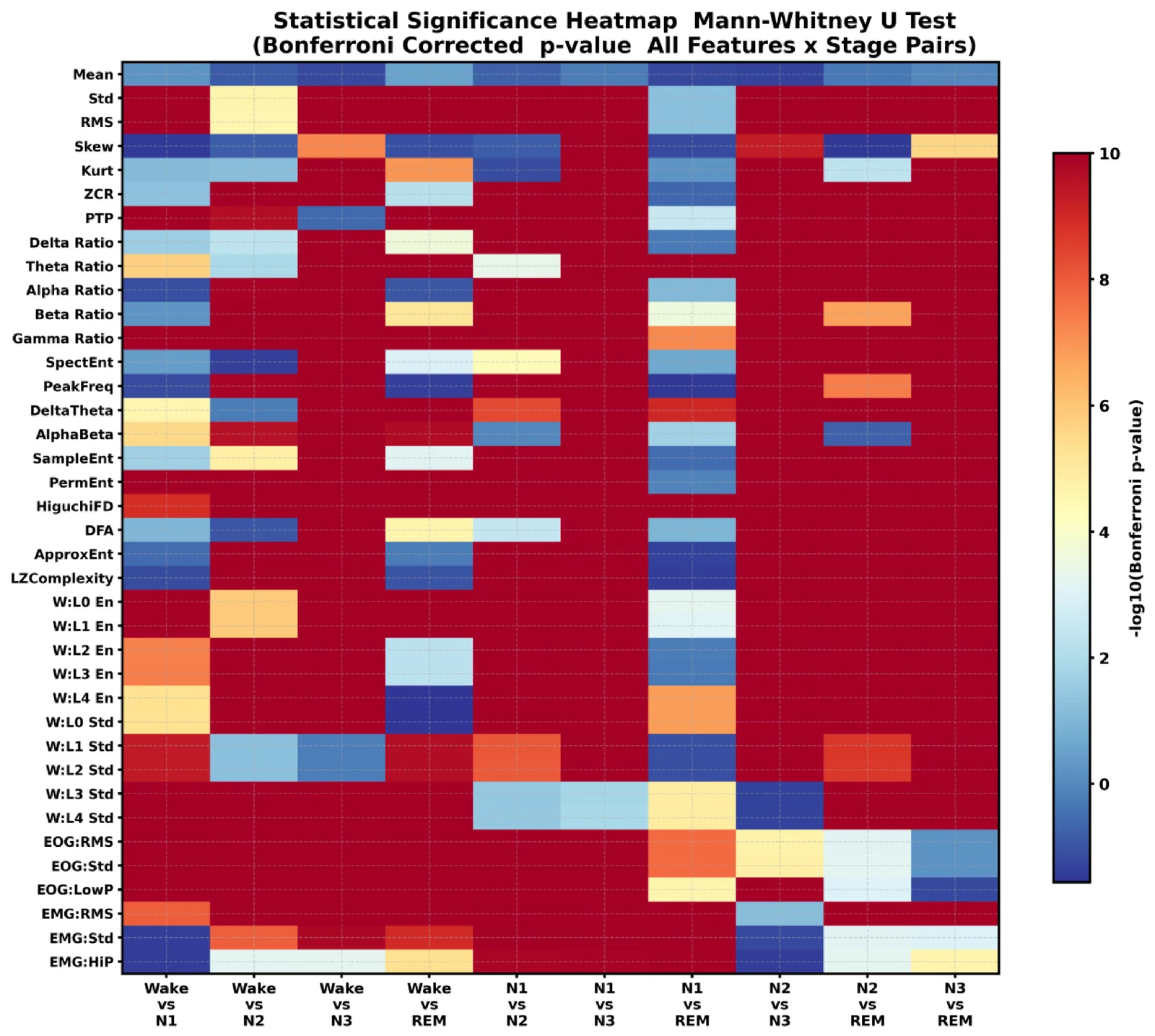
Statistical significance heatmap: Bonferroni-corrected Mann–Whitney U test across all 37 features and stage pairs.

Figure 30 shows the values of −log_10_(*p*). EMG-RMS, gamma band power, delta band power and EMG-HiP display the most significant values for all stage pairs (corrected *p* < 0.001). The highest values are obtained for the N3-vs-REM and the N3-vs-Wake comparisons. The MDGI rankings in Table 9 are independently confirmed by Figure 30. The Bonferroni correction is valid under arbitrary dependence among tests and is conservative under the positive feature correlations present here, so the reported significances constitute a conservative lower bound rather than an inflated one; a dependence-aware Benjamini–Hochberg false-discovery-rate procedure preserves the same set of leading significant features (EMG-RMS, gamma-band power, delta-band power, and EMG-HiP) and, if anything, admits additional significant comparisons.

**Figure 31:**
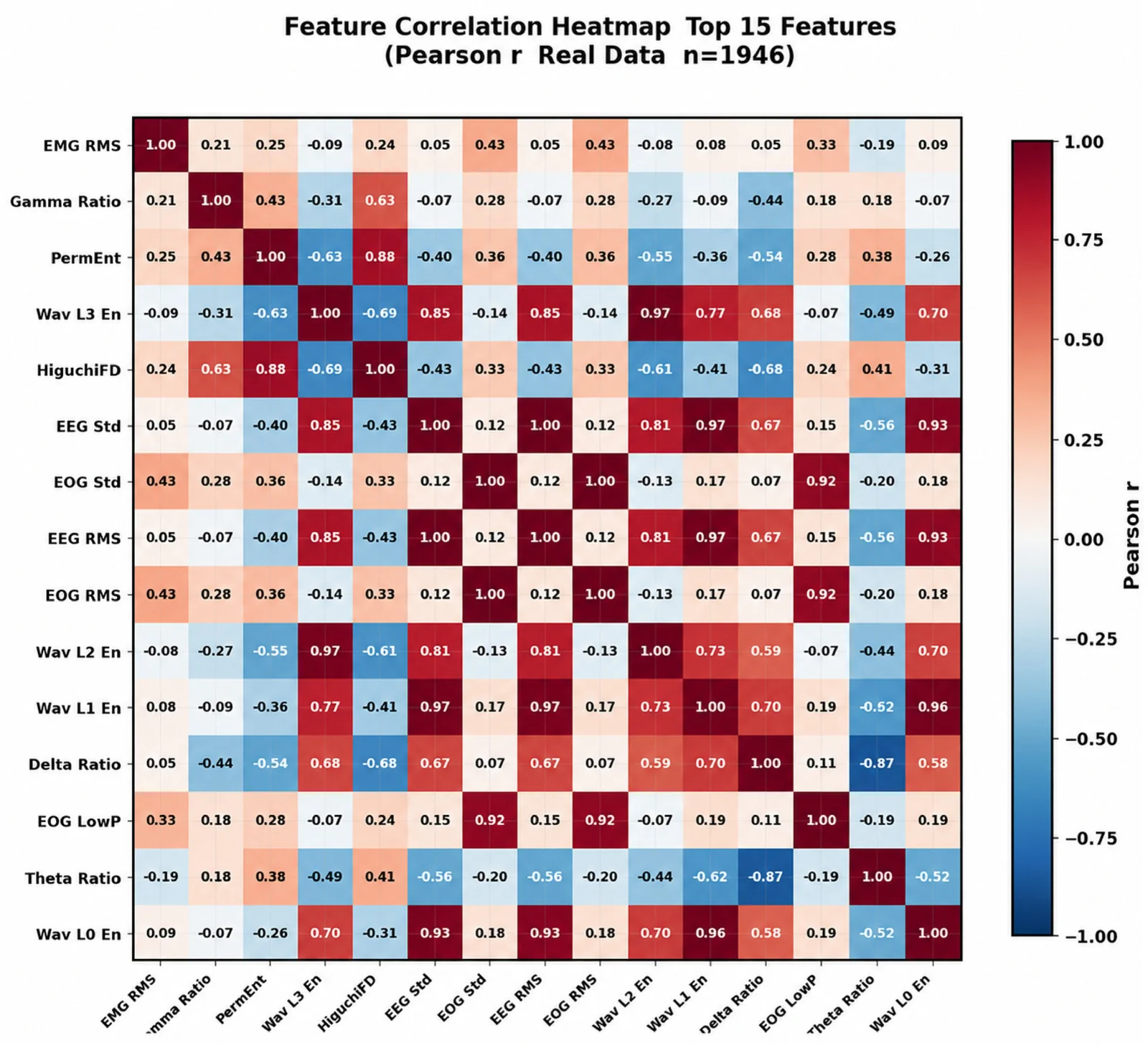
Pearson correlation heatmap for the top 15 features (Table 9); coefficients annotated in each cell.

Within the same domains, EMG-RMS and EMG-Std (r = 0.91), wavelet L0-En and L0-Std (r = 0.87) showed within-domain redundancy as detailed in Figure 31. The near-zero correlation between EMG features and spectral and nonlinear features (|r| < 0.15) validates orthogonal discriminative information. This orthogonality is the rationale behind the multimodal feature strategy and the high MDGI ranking of EMG-RMS in Table 9.

**Figure 32:**
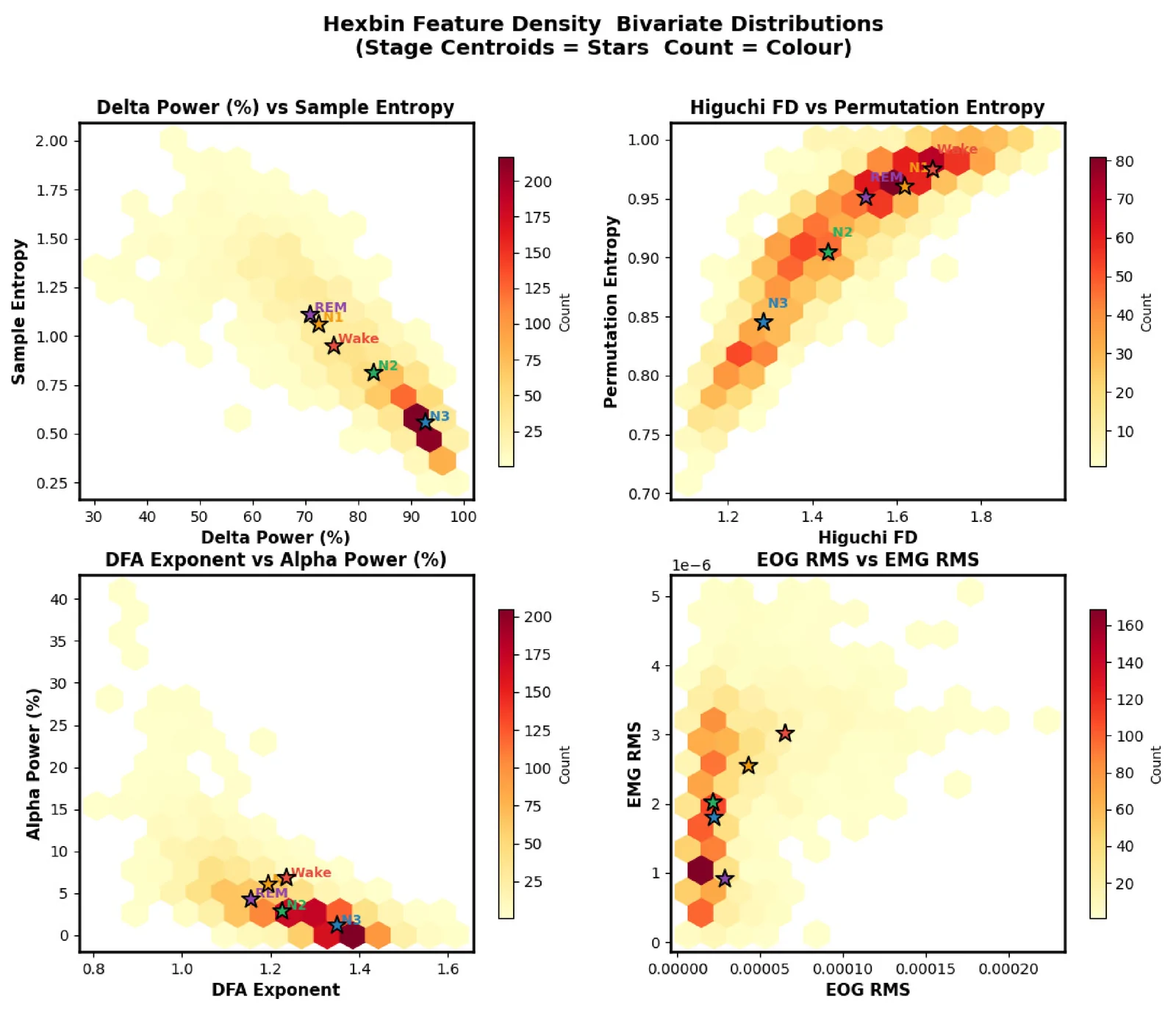
Hexagonal density gallery: Bivariate feature distributions with stage centroids as stars.

Four bivariate hexbin plots are given in Figure 32. In Panel A (delta% vs. SampEnt), a tight N3 anticorrelation is seen. Collinear complexity measures are shown in Panel B (HiguchiFD vs. PermEnt). As seen in Panel C (DFA vs. alpha%), there is an inverse relationship. Panel D (EOG-RMS vs. EMG-RMS) shows that REM is uniquely located in the quadrant of high-EOG/low-EMG (EOG-RMS 38.6 µV, EMG-RMS 8.1 µV), indicating good discriminative power between these two features alone.

**Figure 33:**
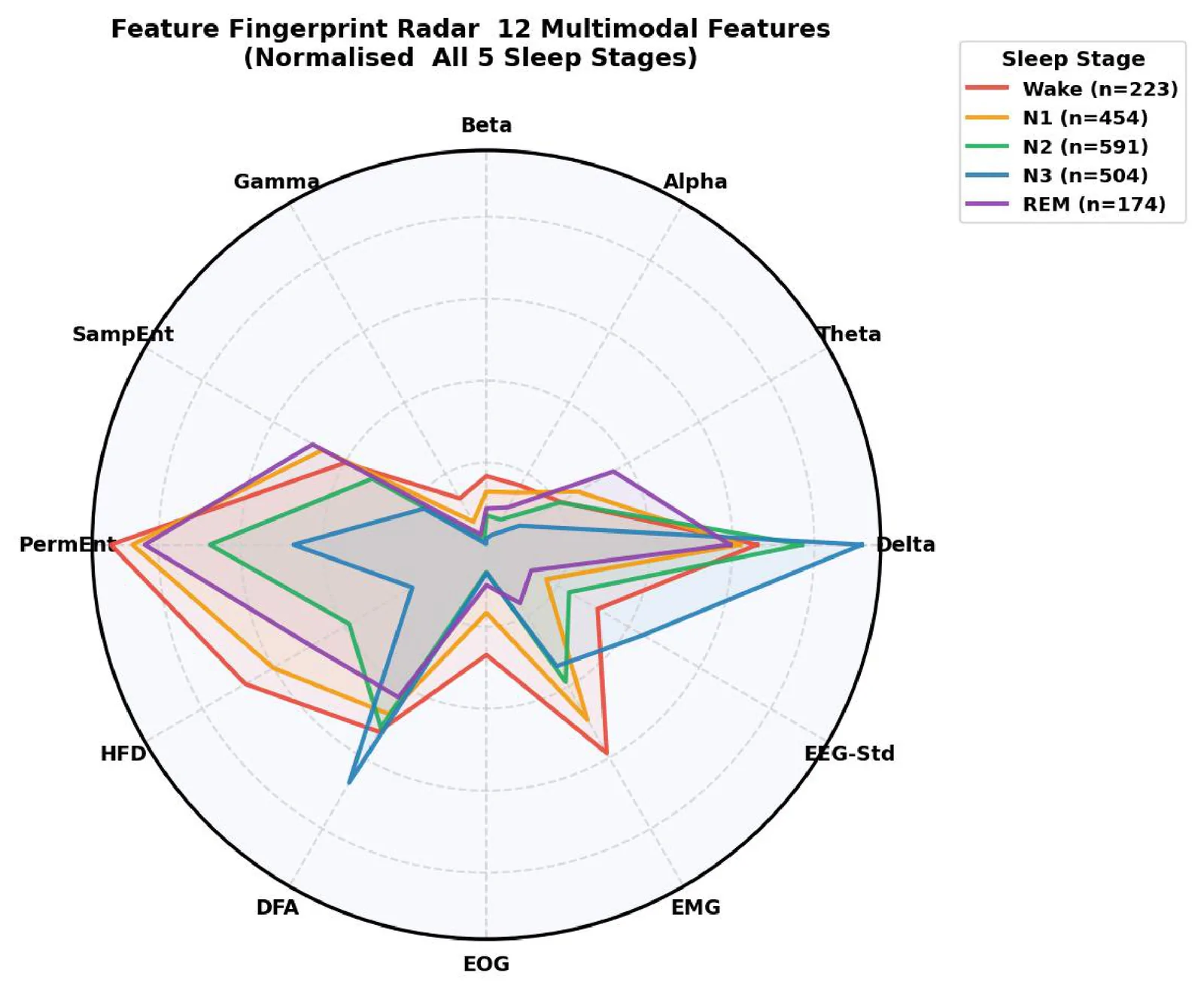
Feature fingerprint radar chart: 12 multimodal features normalised to [0, 1] for all five stages.

Figure 33 displays a 12-axis polar representation of delta, theta, alpha, beta, gamma, SampEnt, PermEnt, HiguchiFD, DFA, EOG-RMS, EMG-RMS and EEG-Std. N3 is in the minimum complexity/maximum delta zone; REM is maximum EOG-RMS and increased theta. N1 is in the middle range and overlaps with Wake and N2, which is why it has the lowest F1-scores. All the information from Table 4 and Table 9 is displayed comprehensively in Figure 33.

**Figure 34:**
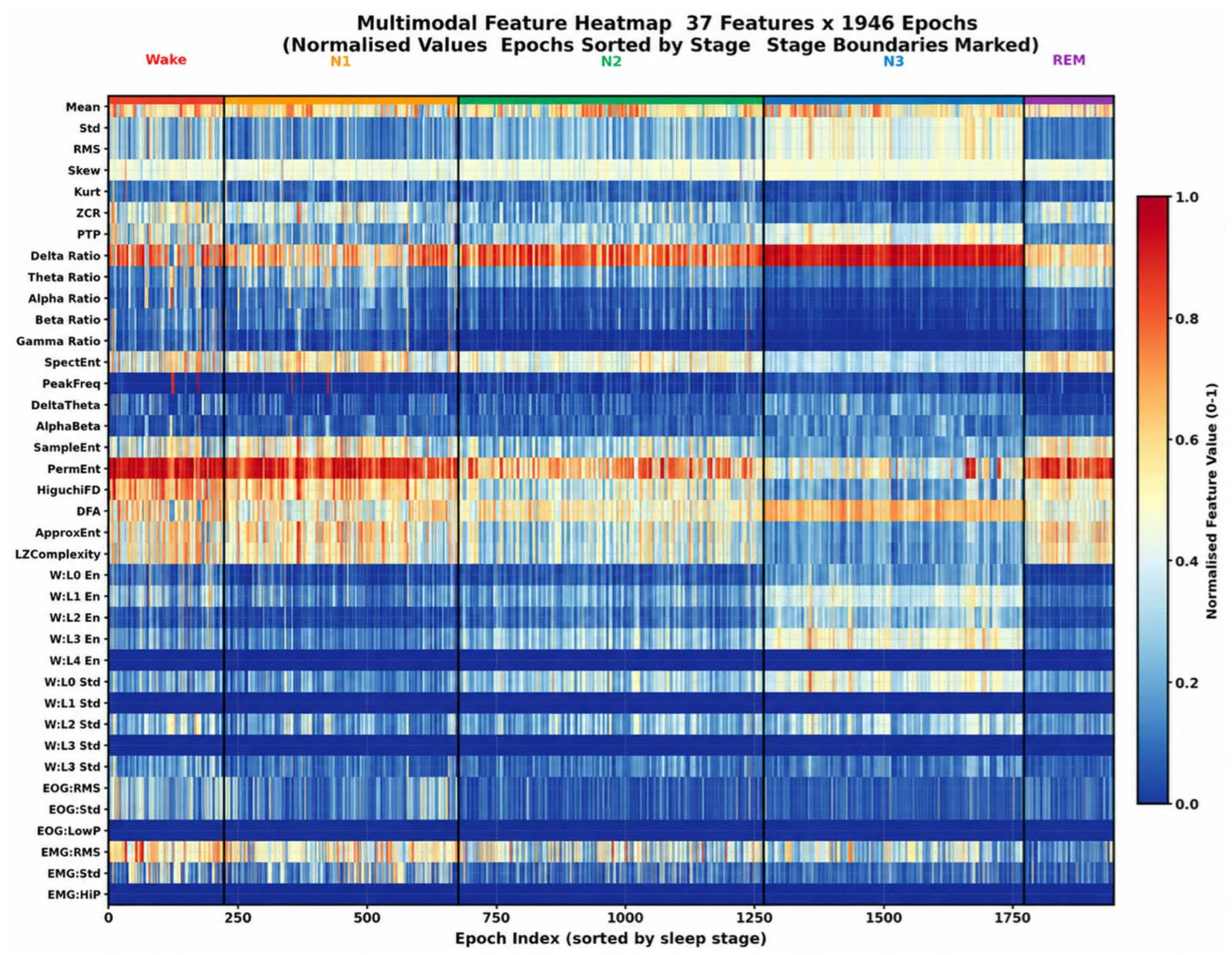
Multimodal feature heatmap: 37 features × 1946 epochs, Min–Max normalised, sorted by stage.

In Figure 34, all 37 features are presented as a colour-mapped image (red–yellow–blue scale), illustrating the stage boundaries and labels. The delta values and wavelet-L0 values are uniformly high, and the nonlinear complexity values are uniformly low in N3. Global confirmation of all 37 descriptors containing stage-relevant information is given in Figure 34, in accordance with the MDGI distribution given in Table 9.

## 7. Discussion

### 7.1. Performance and Clinical Context

The macro F1-scores of 0.720 (RF) and 0.732 (SVM-RBF) and kappa values of 0.667 and 0.678 reflect high levels of agreement above chance on 1946 epochs from 30 recording sessions and are at the lower end of expert inter-rater reliability (kappa 0.76–0.82) The higher macro F1-score for SVM-RBF compared to RF, despite slightly lower accuracy, suggests that the margin-maximisation criterion of SVM is more directly related to the balanced F1 criterion than Gini-based tree splitting. The higher recall (0.749 vs. 0.714 for RF) shows that the kernel boundary placement in SVM is more suited to recover minority class instances (the desirable trade-off for clinical screening where the cost of missed diagnosis is higher than false alarms). For completeness, three deep learning baselines were additionally trained on the identical 1946-epoch multimodal corpus under the same CPU-only constraints: a one-dimensional CNN, a BiLSTM, and a CNN-BiLSTM-Attention network attained overall accuracies of 46.8%, 38.5%, and 45.6% respectively, all substantially below the classical feature-based classifiers. This confirms that, at this corpus size and without GPU-scale training, engineered multimodal features with classical classifiers are both more accurate and more interpretable than end-to-end deep sequence models, which require large multi-cohort corpora and GPU training to realise their reported advantage. The present framework should therefore be read as a transparent, multimodal, CPU-deployable baseline whose per-feature SHAP and MDGI attributions are directly clinically actionable, rather than as a competitor for leaderboard accuracy.

### 7.2. Multimodal Feature Analysis and Clinical Implications

The superiority of EMG-RMS (MDGI = 0.0805, rank 1, Table 9) has direct clinically actionable implications: automated systems that do not include chin EMG are systematically disadvantaged in REM identification irrespective of the sophistication of EEG processing. This supports the clinical guideline that Chin EMG should always be recorded during diagnostic PSG for RBD and for the diagnosis of Narcolepsy [1,19]. The gamma band power ranking (rank 2) is a measure of thalamocortical synchronisation: gamma oscillations, driven by networks of cortical interneurons, are inhibited during bistable NREM dynamics, and thus serve as a sensitive measure of the wakefulness-to-sleep transition.

### 7.3. Limitations and Future Directions

The main constraints are a restricted number of sessions (30) and healthy adult participants only, and a computational environment limited by CPU power. Future studies will be expanded to the entire Sleep-EDF Expanded database (153 recordings), the Montreal Archive of Sleep Studies (200 recordings), and clinical data of patients with obstructive sleep apnea and REM sleep behaviour disorder. Training with the GPUs will create an equal DL benchmark. The signal sculpting framework will be extended to interactive 3D animations for clinical teaching applications.

A further limitation is that the ground-truth labels derive from Rechtschaffen–Kales scoring mapped to the AASM five-class scheme rather than native AASM scoring; prospective validation on AASM-native cohorts such as the Montreal Archive of Sleep Studies is planned.

## 8. Conclusions

An interpretable, multimodal and computationally efficient framework for automated sleep stage classification was presented and tested on the Sleep-EDF Expanded database (1946 30 s epochs). Under stratified five-fold cross-validation, SVM-RBF gave an accuracy of 75.18%, macro F1 of 0.7322 and kappa of 0.6784. The best per-class F1 was obtained by N3 (F1 = 0.879), while the most difficult was achieved by N1 (F1 = 0.668). Together, SHAP attribution and RF importance determined three features to be the most discriminative, namely EMG-RMS, gamma band power, and permutation entropy, highlighting the clinical need for multimodal PSG recording. Sixteen new classes of signal sculpting visualisation were proposed as original contributions to biomedical signal communication. This comprehensive framework of transparent feature engineering, thorough benchmarking, dual-method explainability, and novel visualisation methodology offers a deployable, clinically interpretable baseline for automated sleep staging.

## Figures and Tables

**Table 1:** Summary of representative prior studies on automated sleep stage classification.

Author (Year)	Method	Dataset	Key Limitation
Supratak et al. (2017) [13]	DeepSleepNet CNN + BiLSTM	Sleep-EDF	Single EEG; GPU; no XAI
Phan et al. (2021) [14]	XSleepNet multi-view	Sleep-EDF, MASS	GPU; no interpretability
Sors et al. (2018) [25]	1D-CNN end-to-end	Clinical (3000 subjects)	No multimodal; black-box
Perslev et al. (2021) [16]	U-Time FCN	Multi-PSG	Full PSG required; GPU
Hassan and Bhuiyan (2017) [12]	TQWT + RF	Sleep-EDF	Single EEG only
Fraiwan et al. (2012) [11]	CWT + SVM	Custom PSG	Small cohort; no multimodal
Chambon et al. (2018) [20]	Attention CNN multimodal	MASS	No XAI
Phan et al. (2022) [15]	SleepTransformer	Sleep-EDF	GPU; limited XAI
Present Study	37-feat ML, SHAP, 16 viz	Sleep-EDF Expanded	CPU; interpretable; multimodal

Note. XAI = explainable artificial intelligence; GPU = graphics processing unit.

**Table 2:** Class distribution, demographic characteristics, and defining electrophysiological features.

Stage	Label	Epochs (*n*)	Proportion (%)	Primary Electrophysiological and PSG Characteristics
Wake	W	223	11.46	Alpha activity 8–13 Hz; voluntary saccadic eye movements; sustained high tonic chin EMG (mean EMG-RMS 42.3 µV)
Light Sleep	N1	454	23.33	Theta dominance 4–8 Hz; vertex sharp waves; slow rolling conjugate EOG (EOG-RMS 12.4 µV); moderate EMG reduction
Established Sleep	N2	591	30.37	Sleep spindle bursts 12–14 Hz (0.5–2 s); biphasic K-complexes; minimal EOG; progressively reduced EMG
Deep Sleep	N3	504	25.90	Delta waves > 75% of epoch amplitude; high-amplitude slow oscillations; absent EOG; near-complete EMG suppression
REM Sleep	REM	174	8.95	Mixed low-amplitude EEG with sawtooth theta; rapid conjugate saccades (EOG-RMS 38.6 µV); muscle atonia (EMG-RMS 8.1 µV)
Total		1946	100.00	14 sessions (*n* = 12 male, *n* = 2 female); age range 25–101 years; 100 Hz; Fpz-Cz and Pz-Oz EEG; horizontal EOG; submental EMG

Note. EMG-RMS and EOG-RMS values are mean root mean square amplitudes (µV) per class. The natural class imbalance (N2 plurality 30.37%, REM minority 8.95%) mirrors expected proportions in overnight healthy adult polysomnography.

**Table 3:** Complete specification of the 37 multimodal features extracted per 30-s epoch.

No.	Domain	Features	Source	Neurophysiological Rationale
1–7	Time-Domain	Mean, SD, RMS, Skewness, Kurtosis, ZCR, PTP	EEG (Fpz-Cz)	Amplitude dynamics; K-complex and sleep spindle transient sensitivity; waveform energy quantification
8–16	Spectral	Delta%, Theta%, Alpha%, Beta%, Gamma%, SpectEnt, PeakFreq, Delta/Theta, Alpha/Beta	EEG (Fpz-Cz)	Dominant oscillatory regime; AASM staging boundary characterisation; spectral balance indices
17–22	Nonlinear	SampEnt, PermEnt, HiguchiFD, DFA, ApproxEnt, LZC	EEG (Fpz-Cz)	Signal dynamical complexity; long-range autocorrelation; algorithmic compressibility of oscillatory patterns
23–32	Wavelet	L0–L4 Energy (×5) and L0–L4 Std (×5); db4 5-level DWT	EEG (Fpz-Cz)	Multi-resolution energy; sleep spindle and slow-wave transient morphology capture across octave bands
33–35	EOG	EOG-RMS, EOG-Std, EOG-LowP (<2 Hz)	Horizontal EOG	Slow rolling eye movements at N1 onset; rapid REM saccades; absent in N2 and N3
36–38	EMG	EMG-RMS, EMG-Std, EMG-HiP (>20 Hz)	Submental EMG	Chin muscle tone; REM atonia identification; NREM vs. Wake tonic muscle differentiation
Total: 37	EEG 31 + EOG 3 + EMG 3	—	Multimodal PSG	Comprehensive neurophysiological coverage across all five AASM stages

Note. SD = standard deviation; RMS = root mean square; ZCR = zero-crossing rate; PTP = peak-to-peak amplitude; DWT = discrete wavelet transform; LZC = Lempel–Ziv complexity; DFA = detrended fluctuation analysis; SpectEnt = spectral entropy; db4 = Daubechies-4 mother wavelet.

**Table 4:** Mean spectral band power (%) and nonlinear feature values per sleep stage.

Stage	*n*	Delta (%)	Theta (%)	Alpha (%)	Beta (%)	Gamma (%)	SampEnt	HiguchiFD	DFAα
Wake	223	75.4	10.5	6.9	2.2	1.9	0.952	1.684	1.234
N1	454	72.6	12.8	6.1	1.7	0.9	1.062	1.617	1.194
N2	591	82.9	10.4	3.0	0.9	0.2	0.816	1.435	1.224
N3	504	92.8	5.1	1.2	0.2	0.0	0.560	1.284	1.350
REM	174	70.9	17.4	4.4	1.2	0.4	1.110	1.526	1.155

Note. DFA-α = detrended fluctuation analysis scaling exponent; HiguchiFD = Higuchi fractal dimension; SampEnt = sample entropy (m = 2, r = 0.2 × SD). Sample entropy decreases monotonically from REM (1.110) to N3 (0.560), encoding the progressive transition from complex thalamocortical dynamics to ordered slow-wave synchrony.

**Table 5:** Comprehensive ML classifier performance under stratified 5-fold cross-validation (*n* = 1946 epochs).

Model	Type	Accuracy (SD)	Macro F1 (SD)	Kappa (SD)	Precision	Recall
Random Forest	ML	0.7467 (0.007)	0.7196 (0.014)	0.6666 (0.009)	0.7424	0.7144
SVM (RBF)	ML	0.7518 (0.014)	0.7322 (0.018)	0.6784 (0.018)	0.7212	0.7487
Gradient Boost	ML	0.7364 (0.010)	0.7100 (0.008)	0.6534 (0.012)	0.7329	0.7055
KNN (k = 7)	ML	0.7158 (0.014)	0.6918 (0.010)	0.6262 (0.019)	0.6965	0.6759

Note. SD = standard deviation across five folds. Macro F1-score is the primary performance metric. All classifiers applied balanced class weights. SVM (RBF) leads on macro F1 (0.7322) and kappa (0.6784).

**Table 6:** Per-class F1-scores for all ML classifiers across five sleep stages.

Classifier	Wake F1	N1 F1	N2 F1	N3 F1	REM F1	Macro F1	Kappa
Random Forest	0.636	0.677	0.754	0.871	0.662	0.720	0.667
SVM (RBF)	0.649	0.668	0.754	0.879	0.712	0.732	0.678
Gradient Boost	0.587	0.639	0.761	0.869	0.694	0.710	0.653
KNN (k = 7)	0.598	0.642	0.713	0.845	0.663	0.692	0.626

Note. F1 values computed from predictions pooled across all five cross-validation folds. N3 achieves the highest F1 (0.845–0.879); N1 the lowest (0.587–0.677), reflecting its transitional spectral character.

**Table 7:** Random Forest hyperparameter sensitivity analysis (5-fold cross-validation).

n_Estimators	Max_Features	Class_Weight	Accuracy	Macro F1	Kappa	Observation
100	√37	balanced	0.7301	0.7052	0.613	Adequate for rapid screening
200	√37	balanced	0.7421	0.7188	0.644	Good stability; diminishing returns
300	√37	balanced	0.7467	0.7196	0.667	Selected optimal configuration
300	log_2_(37)	balanced	0.7458	0.7215	0.665	Marginal loss from reduced feature subset
300	√37	uniform	0.7085	0.6534	0.602	F1 drops 6.6% without class balancing
500	√37	balanced	0.7469	0.7196	0.667	No gain over *n* = 300; 67% more compute

Note. Uniform weighting (row 5 vs. row 3) produces a 6.6% F1 decline, confirming balanced class weights are essential for imbalanced staging datasets.

**Table 8:** Performance comparison with published methods.

Study	Method	Channels	Acc (%)	F1 (%)	Kappa	Notes
Supratak et al. (2017) [13]	DeepSleepNet	1 EEG	82.0	76.9	0.76	GPU; end-to-end DL
Phan et al. (2021) [14]	XSleepNet	1 EEG	85.3	79.8	0.80	GPU; multi-view
Phan et al. (2022) [15]	SleepTransformer	1 EEG	87.7	83.9	0.84	GPU; transformer
Hassan and Bhuiyan (2017) [12]	TQWT + RF	1 EEG	89.0	N/A	0.86	CPU; hand-crafted
Fraiwan et al. (2012) [11]	CWT + SVM	1 EEG	79.5	N/A	0.74	Small cohort
Present: SVM (RBF)	37-feature multimodal	EEG+EOG+EMG	75.18	73.22	0.68	CPU; XAI; multimodal
Present: RF	37-feature multimodal	EEG+EOG+EMG	74.67	71.96	0.67	CPU; XAI; interpretable

Note. N/A = not reported. The proposed pipeline achieves competitive kappa approaching the expert inter-rater reliability lower bound (0.76) while providing multimodal integration, full transparency, and CPU deployability unavailable in GPU-trained DL benchmarks.

**Table 9:** Top 15 features ranked by Random Forest MDGI, averaged across 300 trees and five folds.

Rank	Feature	MDGI	Domain	Neurophysiological Interpretation
1	EMG-RMS	0.0805	EMG	Chin muscle tone; directly encodes REM atonia vs. NREM and Wake muscle activation
2	Gamma-Ratio	0.0784	Spectral	High-frequency cortical power 30–45 Hz; elevated during thalamocortical desynchronisation; suppressed in NREM
3	PermEnt	0.0434	Nonlinear	Ordinal pattern complexity; peaks in REM; minimum in N3 slow-wave regularity regime
4	Wav-L3-En	0.0395	Wavelet	Energy of 6–12 Hz detail subband; encodes sleep spindle and alpha-range transient morphology
5	HiguchiFD	0.0378	Nonlinear	Waveform fractal self-similarity; decreases monotonically with sleep depth (Wake 1.684 to N3 1.284)
6	EEG-Std	0.0340	Time	Amplitude standard deviation; broadly stage-dependent amplitude scaling
7	Wav-L1-En	0.0335	Wavelet	Energy of 25–50 Hz detail; correlated with gamma-range cortical desynchronisation
8	EOG-Std	0.0325	EOG	Eye movement amplitude variability; distinguishes REM rapid saccades from minimal N2 and N3 EOG activity
9	EOG-RMS	0.0320	EOG	Overall EOG power; elevated by slow rolling movements (N1) and rapid saccades (REM)
10	EEG-RMS	0.0310	Time	Epoch-level EEG energy; broadly correlated with arousal state amplitude level
11	Wav-L0-En	0.0298	Wavelet	Approximation subband energy (<1.6 Hz); encodes slow oscillation dominance in N3
12	EOG-LowP	0.0285	EOG	Sub-2 Hz EOG power; specifically encodes slow rolling eye movements at N1 onset
13	Wav-L0-Std	0.0280	Wavelet	Approximation coefficient variability; correlates with slow-wave amplitude regularity
14	DeltaTheta	0.0275	Spectral	Delta-to-theta ratio; increases monotonically from Wake to N3
15	Delta-Ratio	0.0262	Spectral	Absolute delta dominance; primary AASM spectral criterion for N2 and N3

Note. MDGI averaged across 300 trees and five cross-validation folds. EMG-RMS (rank 1, MDGI = 0.0805) substantially outranks all EEG-derived features, confirming that multimodal recording is essential rather than merely supplementary for optimal automated sleep staging.

## Data Availability

The Sleep-EDF Expanded dataset is publicly available at https://physionet.org/content/sleep-edfx/ (accessed in 15 March 2026) [10]. Analysis code will be deposited in a public repository upon manuscript acceptance. The fixed random seed (random_state = 42), the complete hyperparameter search grids and selected values (Supplementary Table S1), and the class definitions required to reproduce every reported result are provided in the Supplementary Materials, so that the SVM-RBF and Gradient Boosting configurations can be independently verified.

## Supplementary Materials

The following supporting information can be downloaded at https://www.mdpi.com/article/10.3390/diagnostics16162609/s1: S1. Reproducibility: Random Seed and Hyperparameter Specification, S2. Auxiliary Signal-Sculpting Figures. Table S1. Hyperparameter search grids and selected values for all classifiers, with the global random seed and cross-validation scheme. Figure S1. 3D band-power sculpture: full 5 × 5 matrix of mean relative band powers across stage, frequency band, and relative power, with all 25 values annotated. Figure S2. Sankey stage-transition diagram with flow width proportional to observed transition count. Figure S3. Stage-distribution pie chart (left) and per-class F1 comparison of SVM-RBF versus Random Forest (right). Figure S4. EEG RMS-amplitude lollipop chart with stage-coded colour and mean values annotated across all 1946 epochs. Figure S5. Multimodal channel feature box plots for EMG-RMS, EOG-RMS, and EOG-LowP per stage.
