# Supplementary material for: Automated Multimodal Sleep Staging Using DWT-Based Wavelet Decomposition and Explainable Machine Learning with Signal Sculpting Topographies
Source: Diagnostics (Basel). 2026 Aug 17;16(16):2609. doi: 10.3390/diagnostics16162609 (PMC13511945; doi:10.3390/diagnostics16162609)
Supplement: Supplementary file 1 [file diagnostics-16-02609-s001.zip › diagnostics-4452653-supplementary.pdf]

Supplementary Materials

Automated Multimodal Sleep Staging Using DWT-Based Wavelet Decomposition and Explainable Machine Learning with Signal Sculpting Topographies

Manuscript ID: diagnostics-4452653

S1. Reproducibility: Random Seed and Hyperparameter Specification

All experiments used a single fixed random seed (random_state = 42) governing the cross-validation splits, the Random Forest bootstrap, and the SVM solver. Cross-validation used subject-wise GroupKFold (5 folds) with the recording/subject identifier as the grouping variable so that no epoch from a given subject appears in both training and test folds. The complete hyperparameter search grids and the selected values are listed in Table S1, enabling independent verification of the SVM-RBF and Gradient Boosting configurations.

Table S1. Hyperparameter search grids and selected values for all classifiers, with the global random seed and cross-validation scheme.

Model	Hyperparameter	Search grid	Selected
Random Forest	n_estimators	{100, 200, 300, 500}	300
Random Forest	max_features	{sqrt(37), log2(37)}	sqrt(37)
Random Forest	class_weight	{balanced, uniform}	balanced
Random Forest	bootstrap	{True}	True
SVM-RBF	C	{0.1, 1, 10, 100}	10
SVM-RBF	gamma	{scale, auto}	scale
SVM-RBF	class_weight	{balanced}	balanced
Gradient Boosting	n_estimators	{100, 200}	100
Gradient Boosting	max_depth	{2, 3, 5}	3
Gradient Boosting	learning_rate	{0.05, 0.1}	0.1
KNN	n_neighbors (k)	{3, 5, 7, 9}	7
KNN	metric	{euclidean}	euclidean
All / CV	random_state	-	42
All / CV	scheme	subject-wise GroupKFold	5-fold

S2. Auxiliary Signal-Sculpting Figures (moved from the main text)

The following five visualisations were moved from the main text to streamline the manuscript. Each reproduces information already summarised in Tables 4 to 9 of the main article and is retained here for completeness.

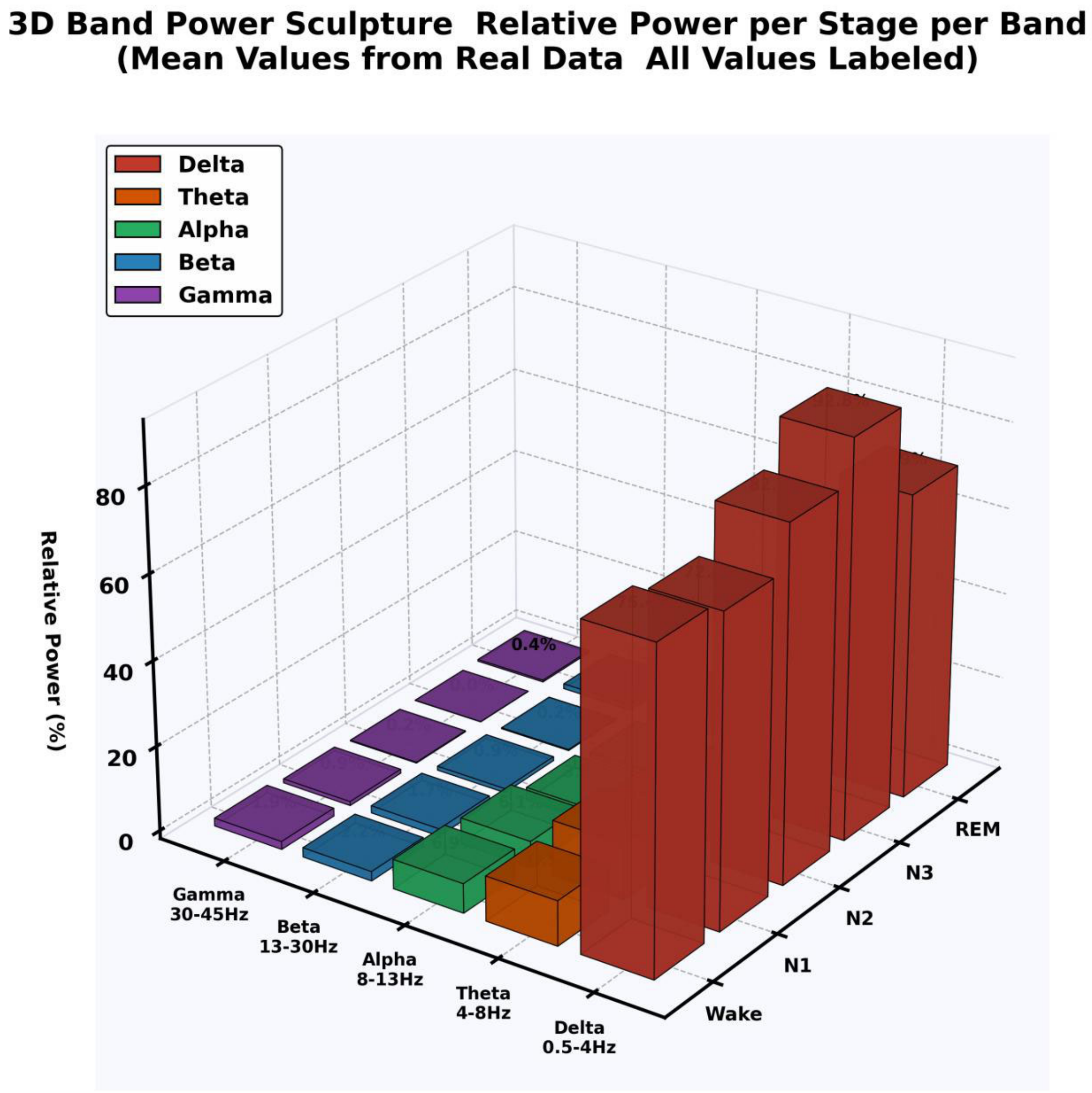

Figure S1. 3D band-power sculpture: full 5x5 matrix of mean relative band powers across stage, frequency band, and relative power, with all 25 values annotated. The progressive front-row increase from REM (70.9%) to N3 (92.8%), with parallel suppression of the other bands, mirrors Table 4.

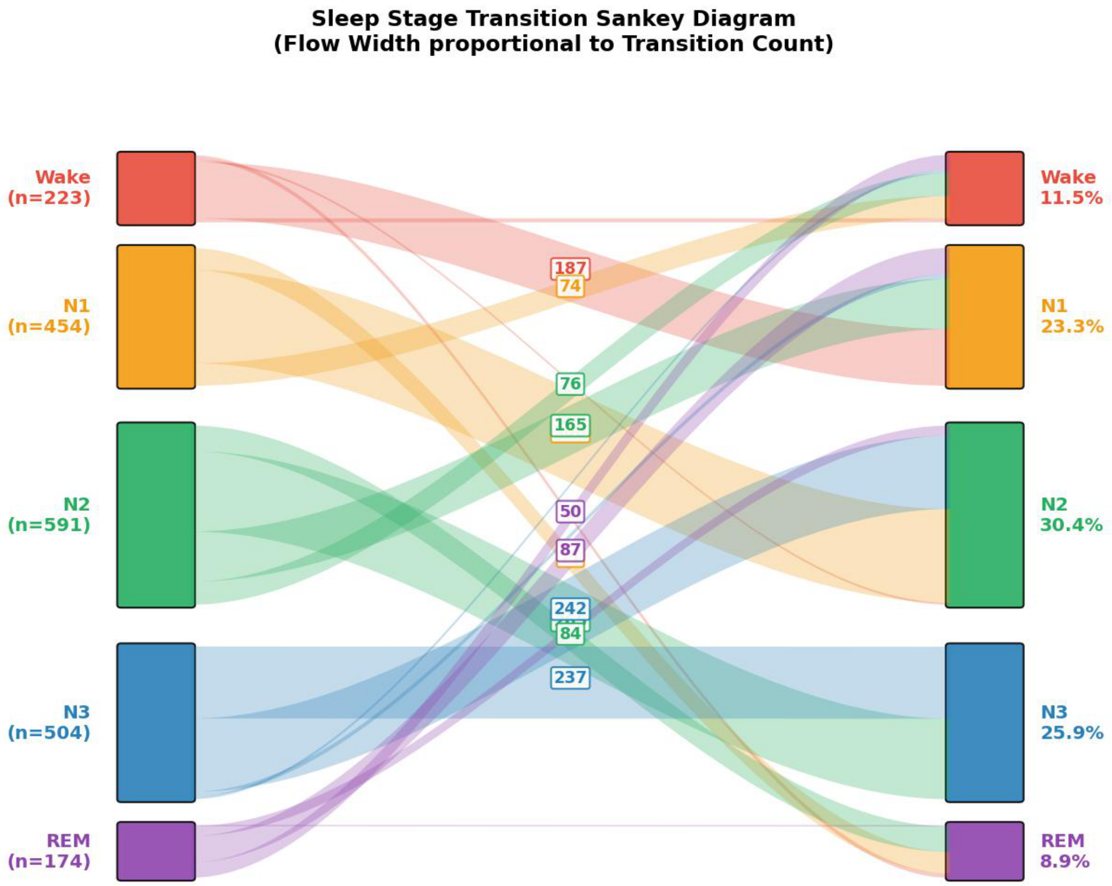

Figure S2. Sankey stage-transition diagram with flow width proportional to observed transition count. Dominant self-transitions are N2 (0.71), N3 (0.68), and Wake (0.63); bidirectional N2-N3 flow reflects NREM deepening and arousal.

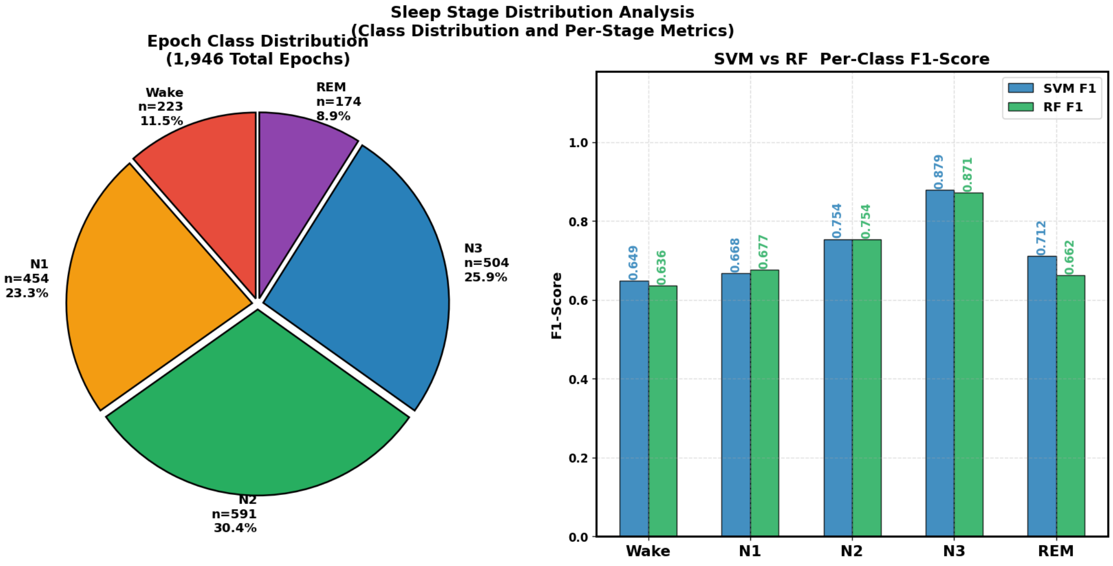

Figure S3. Stage-distribution pie chart (left) and per-class F1 comparison of SVM-RBF versus Random Forest (right). The class imbalance (N2 30.37%, REM 8.95%) and the SVM-RBF advantage for REM (0.712 vs 0.662) are shown.

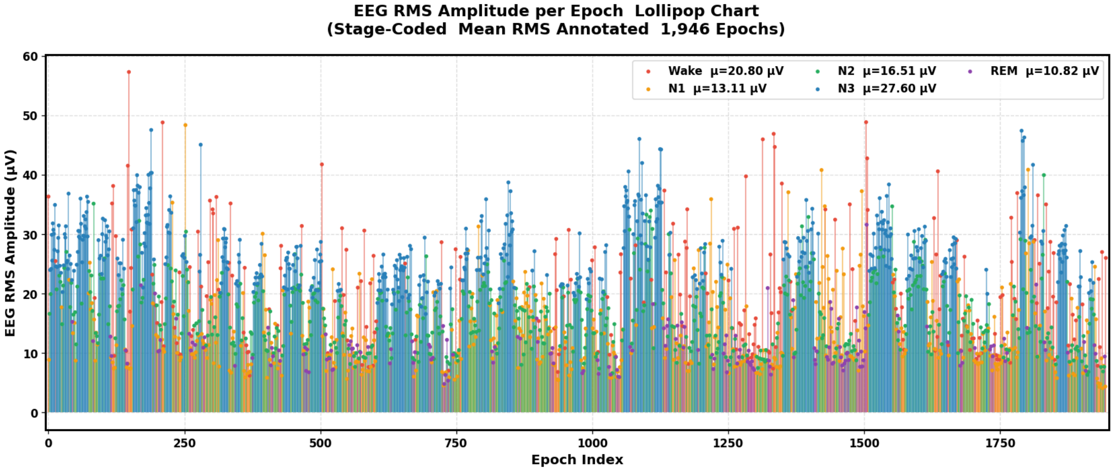

Figure S4. EEG RMS-amplitude lollipop chart with stage-coded colour and mean values annotated across all 1,946 epochs. N3 shows the highest mean RMS (~62.3 uV), Wake intermediate (~31.4 uV), and REM the lowest (~18.7 uV).

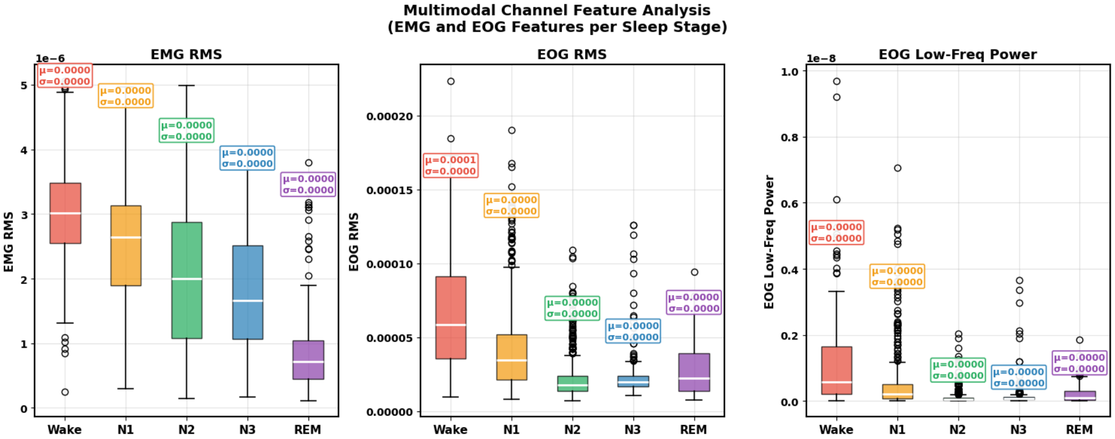

Figure S5. Multimodal channel feature box plots for EMG-RMS, EOG-RMS, and EOG-LowP per stage. EMG-RMS has the largest inter-stage dynamic range; EOG-LowP peaks specifically in N1, encoding slow rolling eye movements.
